# Connecting Top-Down and Bottom-Up Approaches in Environmental Observing

**DOI:** 10.1093/biosci/biab018

**Published:** 2021-04-28

**Authors:** Hajo Eicken, Finn Danielsen, Josephine-Mary Sam, Maryann Fidel, Noor Johnson, Michael K Poulsen, Olivia A Lee, Katie V Spellman, Lisbeth Iversen, Peter Pulsifer, Martin Enghoff

**Affiliations:** University of Alaska Fairbanks, Fairbanks, Alaska, United States; University of Alaska Fairbanks, Fairbanks, Alaska, United States; University of Alaska Fairbanks, Fairbanks, Alaska, United States; Yukon River Inter-Tribal Watershed Council, Anchorage, Alaska, United States; University of Colorado, Boulder, Boulder, Colorado, United States; University of Alaska Fairbanks, Fairbanks, Alaska, United States; University of Alaska Fairbanks, Fairbanks, Alaska, United States; University of Alaska Fairbanks, Fairbanks, Alaska, United States; Nansen Environmental and Remote Sensing Center, Bergen, Norway; Carleton University, in Ottawa, Ontario, Canada; University of Alaska Fairbanks, Fairbanks, Alaska, United States

**Keywords:** community-based monitoring, observing systems, citizen science, comanagement, coproduction of knowledge

## Abstract

Effective responses to rapid environmental change rely on observations to inform planning and decision-making. Reviewing literature from 124 programs across the globe and analyzing survey data for 30 Arctic community-based monitoring programs, we compare top-down, large-scale program driven approaches with bottom-up approaches initiated and steered at the community level. Connecting these two approaches and linking to Indigenous and local knowledge yields benefits including improved information products and enhanced observing program efficiency and sustainability. We identify core principles central to such improved links: matching observing program aims, scales, and ability to act on information; matching observing program and community priorities; fostering compatibility in observing methodology and data management; respect of Indigenous intellectual property rights and the implementation of free, prior, and informed consent; creating sufficient organizational support structures; and ensuring sustained community members’ commitment. Interventions to overcome challenges in adhering to these principles are discussed.

Local to global-scale transformational change in socioenvironmental systems (Steffen et al. 2018) requires responses that build on informed governance, planning, and decision-making. To overcome challenges and to benefit from opportunities associated with such transitions, society needs to observe, track, understand, and predict environmental change. Observing and monitoring efforts that capture socioenvironmental system behavior across relevant spatiotemporal scales are key in this context. Large-scale programs or high-level frameworks, often driven by governmental action and referred to in the present article as top-down approaches (see box 1 for term definitions), are making great strides toward more coordinated, networked activities. Examples include the Group on Earth Observations (GEO) Global Earth Observing System of Systems (GEOSS; Lautenbacher 2006) and the Global Ocean Observing System (GOOS, Lindstrom et al. 2012). At the same time, community-based monitoring (CBM), a bottom-up approach if initiated and steered at the local level (see box 1), is expanding rapidly (Kouril et al. 2015, Pocock et al. 2018).

**Box 1. Glossary.**

**Bottom up.** Observing or monitoring efforts defined and undertaken at the local scale and brought forward to higher-level bodies, often with a focus on supporting outcomes desired by a local community.

**Community.** A group of people who share a place and an environment or institution. In this collection of articles, we equate community with a local geographical scale that encompasses the range over which individuals travel pursuing resource-harvesting activities (see figure 5).

**Community-based monitoring.** Similar to community-driven monitoring but also including observing and monitoring activities undertaken by community members that are led or defined by noncommunity members, such as scientists or agency personnel. If not driven by the local community, it is possible that such community-based monitoring may represent a top-down (e.g., NASA's GLOBE program, Spellman et al. 2018) as opposed to a bottom-up approach.

**Community data.** Data that a particular community, or group acting as stewards and representatives of the community, has a specific interest in maintaining. Often, residents have had a role in producing this data; sometimes the data may be about a community or its members. For reasons such as sensitivity of content, the community may cultivate a sense of ownership and responsibility on the part of community members.

**Community-driven or community-led monitoring or observing.** A process of routinely observing or monitoring environmental or social phenomena, or both, which is led and undertaken by community members and can involve external collaboration and support of visiting researchers and government agencies (Johnson et al. 2015). Community-driven and -led monitoring is exemplary of bottom-up approaches.

**Coproduction.** In this collection of articles *coproduction* is used narrowly to describe joint generation of new knowledge in the context of resource management, sustainability science, and adaptation to rapid change. It is a joint effort between classically trained scientists (in academia, government agencies, or civil society organizations), holders of Indigenous or local knowledge, or government agencies (including tribal governments). In the context of observing or monitoring programs, coproduction may include codesign of observing systems, and comanagement of observing and systems operations.

**Indigenous knowledge.** Understandings, skills, and worldviews developed by societies with centuries to millennia of interactions with their natural surroundings, and with potential to inform decision-making about fundamental aspects of day-to-day life. This knowledge is integral to a cultural context that includes language, systems of classification, resource use practices, social interactions, rituals, and spirituality (modified from UNESCO 2019 and ICC 2016). Indigenous knowledge is highly diverse and evolves continuously through interaction of experiences, innovations, and various types of knowledge (written, oral, visual, tacit, gendered, practical, and scientific). Most Indigenous and local knowledge systems are empirically tested, applied, contested, and validated through different means in different contexts (Hill et al. 2020).

**Interoperability.** The properties of data and information systems, devices, and applications, which allow them to interact and share with other information products or systems within and across organizational boundaries to provide rapid and seamless information portability (based on HIMSS 2019).

**Local knowledge.** Skills and understandings developed by groups of individuals in a specific local geographic setting, often informing decision-making in day-to-day life. In contrast with Indigenous knowledge, local knowledge does not presuppose a broader, shared worldview, although it often is associated with a shared local understanding of context. Most local and Indigenous knowledge systems are empirically tested, applied, contested, and validated through different means in different contexts (Hill et al. 2020).

**Locally based monitoring.** A broad range of approaches, from self-monitoring of harvests by local resource users themselves, to censuses by local rangers, and inventories by amateur naturalists; we include techniques labeled as *participatory monitoring, community-based monitoring, hunter self-monitoring, and ranger-based monitoring*. Many of these approaches are directly linked to resource management, but the entities being monitored vary widely, from individual animals and plants, through habitats, to ecosystem goods and services. However, all of the approaches have in common that the monitoring is carried out at a local scale by individuals with little formal education, and that local people or local government staff are directly involved in data collection and (in most instances) analysis (Danielsen et al. 2005).

**Monitoring.** Tracking of a particular variable or phenomenon over time, with an eye toward identifying trends that require some type of action—for example, for adaptation or for resource management.

**Observing.** Recording data for a particular variable or process, often in the context of scientific research programs devoted to understanding and predicting the behavior of a particular system. In the context of Indigenous cultures, observing is an awareness and attentiveness to the environment and changes in the environment over time, and is conducted as part of activities associated with daily life such as harvesting, preparing food, and making clothing or other items from harvested materials.

**Top down.** Observing or monitoring efforts defined within the context of a global, international, or national framework, often with a focus on national and international assessments and scientific research; top-down approaches typically define essential variables that link to broad societal benefits and more specific agency or operational missions (see figure 2).

Linking top-down and bottom-up activities may provide substantial benefits, including the ability to tie improved understanding and prediction of large-scale socioenvironmental systems directly to management outcomes desired by local-scale actors. Local-scale outcomes often depend on intertwined processes that relate to a complex set of drivers—for example, those governing community health and social and economic sustainability (figure 1). Large-scale observing systems typically focus on isolating a narrow set of variables that are then tracked uniformly. Often, they employ satellite remote sensing or autonomous sensor systems to help understand and predict system behavior, such as essential climate variables tracked by GEOSS or GOOS (figures 1 and 2). Linking top-down and bottom-up approaches therefore also contributes to complementarity and fit between locally based monitoring (see box 1) most relevant to decision-makers and community planners and large-scale observations. Linking approaches also could enable effective transitioning of scientist-driven research observations into operational monitoring programs that inform action and contribute to sustaining observing and monitoring efforts over time. In this context, a key question emerges: What are major benefits, challenges, and possible interventions to better connect top-down and bottom-up approaches in environmental monitoring?

**Figure 1.**
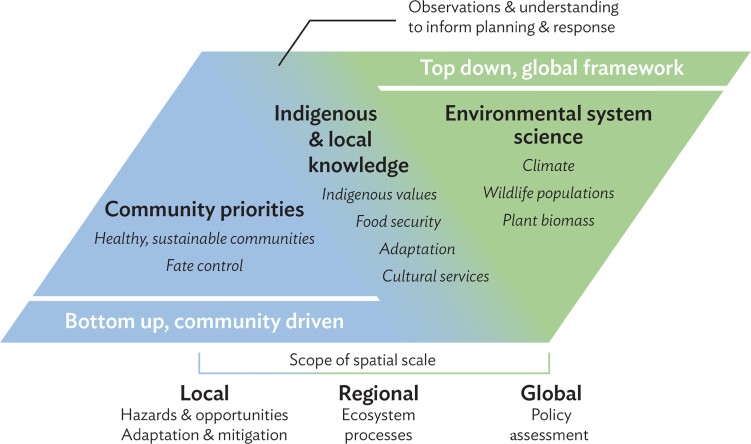
Observing scales and priorities for top-down approaches embedded in international frameworks and focused on indicators and assessments or projections of system state, and bottom-up, community-driven approaches initiated and steered within the local community and focused on outcomes desired by community members. Indigenous and local knowledge inform community-driven approaches but also may serve as a bridge between approaches and scales.

**Figure 2.**
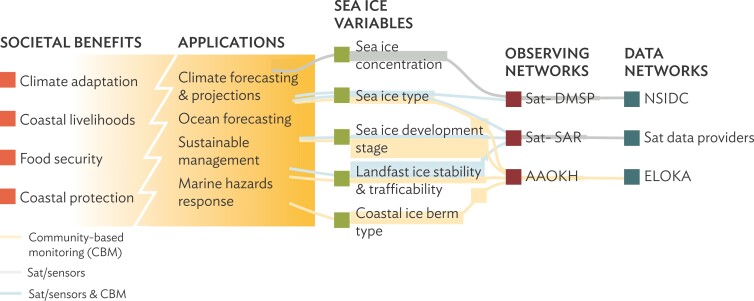
Example of observing network design and implementation viewed through the lens of a top-down, global observing framework approach (Starkweather et al. 2018). In this figure the GOOS Framework on Ocean Observing (Lindstrom et al. 2012) has been applied to Arctic sea-ice observations. Definition of the upper three sea ice variables was through a top-down, scientist-driven process that interfaced with mission agencies (e.g., national ice and climate services) for specific applications such as shipping or large-scale climate assessments. Note that some sea ice variables serve local community information needs, in particular if collected through CBM efforts and at scales relevant to community ice uses, illustrated by the lower two variables that build on CBM efforts through the Alaska Arctic Observatory and Knowledge Hub (AAOKH) and the Exchange for Local Observations and Knowledge of the Arctic (ELOKA). Abbreviations: DMSP, Defense Meteorological Satellite Program; SAR, synthetic-aperture radar; NSIDC, National Snow and Ice Data Center.

We address these questions by analyzing three sources of information: a literature review of CBM programs initiated and implemented by communities or designed by outsiders to address community needs across the globe (table 1), a review of the Arctic CBM literature (Johnson et al. 2016), and an analysis of self-assessments of Arctic CBM programs (Danielsen et al. 2020). The focus on the Arctic is motivated by the potential of findings from the high North to inform broader development of good practices in linking CBM to large-scale, top-down observing system design. The Arctic holds a disproportionate fraction of Earth's coastlines, shelf seas, and terrestrial wetlands; it figures prominently in the life cycles of migratory species; and it provides ecosystem services to Arctic Indigenous peoples and the global community (Eicken et al. 2009, Lenton 2012). Arctic socioenvironmental systems are undergoing transformational, rapid changes exceeding those in other regions, largely as a result of feedback processes involving snow, ice, and permafrost (Lenton 2012). The extent and rate of change experienced in northern regions require responses that span scales from the community to the regional and international level, offering insights relevant for other parts of the globe. The high degree of connectivity between different subsystems and sectors of human activity in the Arctic also has fostered pragmatic approaches to building monitoring programs to support adaptation, mitigation, and innovation.

**Table 1.** The number of studies of community-based monitoring programs initiated and implemented by the communities (bottom up), designed by outsiders to address perceived community needs (top down), and incorporating Indigenous and local knowledge as identified in a global literature review and in a self-reported survey of Arctic programs.

Region	Number of studies	Bottom up	Top down	Bottom up and top down	ILK
Literature review					
Arctic	24	19	5	0	8
Mid-latitudes	42	20	19	0	4
Tropics	51	21	29	1	5
Worldwide	18	6	13	1	3
Self-reported					
Arctic	30	9	10	2	16

*Note:* Some programs covered more than a single region; therefore, the sum of studies referenced in the second column for the literature review (*n* = 135) exceeds the total number of studies analyzed (*n* = 124). Some studies did not fit in to the bottom-up and top-down categories. See box 1 for definitions. *Abbreviation:* ILK, Indigenous or local knowledge.

## Top-down and bottom-up observing and monitoring

Top-down observing or monitoring by large-scale programs, often informed by high-level frameworks, typically derives observing system requirements from scientific research programs or government agency directives at the national or international level. GEO's Global Agricultural Monitoring Activity is an example of this approach. Observing system requirements for this effort to track crop status and provide global-scale agricultural outlooks are determined through the GEO consortium and the Committee on Earth Observation Satellites (Whitcraft et al. 2015). Scientific experts primarily within academia and government agencies determine target variables and guide implementation of the monitoring network, referencing broad Societal Benefit Areas and specific missions defined under GEO.

Historically, many such observing efforts have grown out of research programs that transitioned into operational monitoring systems that are pushed by technological and scientific advances and pulled by information needs of decision-makers (e.g., the Pacific Tropical Atmosphere Ocean Array, McPhaden et al. 2010, or the Circumpolar Biodiversity Monitoring Program, CBMP, Gill and Zöckler 2008). Any environmental monitoring program faces the question of how to ensure that observations support planning and decision-making while generating tangible societal benefits. Typically, top-down observing programs establish such connections through broad-based analysis of benefits using a range of approaches, such as value-tree analysis (IDA STPI and SAON 2017), economic valuation (Dobricic et al. 2018), or integrated environmental modeling and assessments (Laniak et al. 2013).

In contrast, bottom-up approaches originate at the local community level, with observing requirements tied to management outcomes that community members or institutions are seeking to achieve (figure 1; Danielsen et al. 2009, Commodore et al. 2017). Achieving specific goals or management outcomes—particularly in light of environmental change or changing human practices in the region—are at the heart of bottom-up efforts. Members of the scientific research community also may be part of such bottom-up approaches—notably, in participatory action research (Ison 2008).

Examples of bottom-up approaches include coastal marine resource management in Oceania (Johannes 2002) and Greenland (Danielsen et al. 2014a); observations, analysis, and interpretation of disruptive events relevant to wildlife management in Namibia (Stuart-Hill et al. 2005); and the use of community-developed metrics by Maori in New Zealand to track forest health (Timoti et al. 2017). Bottom-up monitoring shares a combination of attributes relating to local initiative and control in establishing the observations, and community involvement in data acquisition and analysis (Danielsen et al. 2014b). Bottom-up monitoring activities have long been part of aboriginal subsistence harvest practices and are increasingly part of comanagement agreements (Huntington 1992).

Top-down and bottom-up monitoring each face significant challenges. A particular problem for top-down monitoring is the potential disconnect between societal benefits and the observing and data distribution networks (figure 2). Top-down approaches are often identified by scientists and agency personnel and may draw only indirectly on input from data users, without clear prioritization or ranking. This problem presents challenges in implementing and sustaining monitoring programs in support of international environmental agreements and treaty systems (Danielsen et al. 2014b). In the Arctic and the sub-Arctic, accounting for roughly half of the world's wetlands and a third of the world's ocean shelves (CAFF 2013), the Arctic Council's CBMP has made great strides in establishing a biodiversity assessment process that draws on monitoring of indicator variables (CAFF 2013). However, the monitoring framework is largely a top-down effort informed by global frameworks (such as the Convention on Biodiversity), with only one out of eight indicator selection criteria referencing information needs of Arctic communities and policymakers (Gill and Zöckler 2008). Bottom-up initiatives that are responsive to the local situation (Danielsen et al. 2017) or focused on specific management outcomes—such as a food security framework put forward by the Inuit Circumpolar Council Alaska (ICC 2015)—may help to maintain relevance and facilitate continuity of monitoring programs. At the same time, the potential of bottom-up approaches to contribute data to global observing programs often remains unrealized because of lack of capacity for local audiences to engage with activities and frameworks beyond the local scale. Blending the two approaches may enhance outcomes and contribute to greater resilience and sustainability of local-scale efforts.

## Community-based environmental monitoring: Perspectives from a review of the global literature

To obtain a broader perspective on the relative proportion of top-down and bottom-up approaches in CBM, and to ­identify promising mechanisms to connect the two, we reviewed the literature on environmental monitoring across the globe. The Scopus database yielded 124 relevant peer-reviewed articles for the time period 1998–2019 that were focused on community-based environmental monitoring studies (table 1; also see the supplemental material on methodology and annotated references). For this review, we distinguish among monitoring efforts confined to Arctic and similar sub-Arctic ecoregions, midlatitudes, and tropical regions, as well as those that cover multiple regions of the world (table 1). On the basis of author-provided keywords and text content, CBM activities were categorized as top down, bottom up, or both. Drawing on Commodore and colleagues (2017), we characterize bottom-up community-based environmental-monitoring programs as initiated, implemented, and managed by the communities. Top-down programs are typically designed by outsiders to address perceived community needs (Danielsen et al. 2009, Seak et al. 2012). We also noted whether CBM activities incorporated Indigenous or local knowledge. Such links can substantially enhance benefits and outcomes associated with a CBM program.

Overall, Arctic programs are represented disproportionately by a factor of more than two relative to programs in the tropics and midlatitudes on the basis of the geographic area covered by the different regions. Furthermore, the preponderance of bottom-up approaches in the Arctic compared with other regions is noteworthy (Moussy et al. 2021). Regional contrasts tied to economics, postcolonialism, and resource access may explain part of this difference (Moussy et al. 2021). Holck (2008) notes that limited funds and competing needs make it difficult for developing countries to establish monitoring programs. Therefore the most viable monitoring schemes are usually funded and implemented by international agencies but are short lived because of the inability of local governments to sustain them. Community-level observing action is often driven by environmental and socioeconomic drivers associated with rapid change (Nakashima et al. 2012). In the Arctic, these drivers are often linked, such as in Nunavik, Canada, where impending rare earth element mining in conjunction with climate change has motivated the establishment of a community watershed monitoring program (Gérin-Lajoie et al. 2018). Similarly, in Arctic coastal environments, the loss of sea-ice habitat and increasing maritime and resource development activities are of particular concern and have led to CBM efforts that support adaptation and response (Johnson et al. 2016).

**Figure 3.**
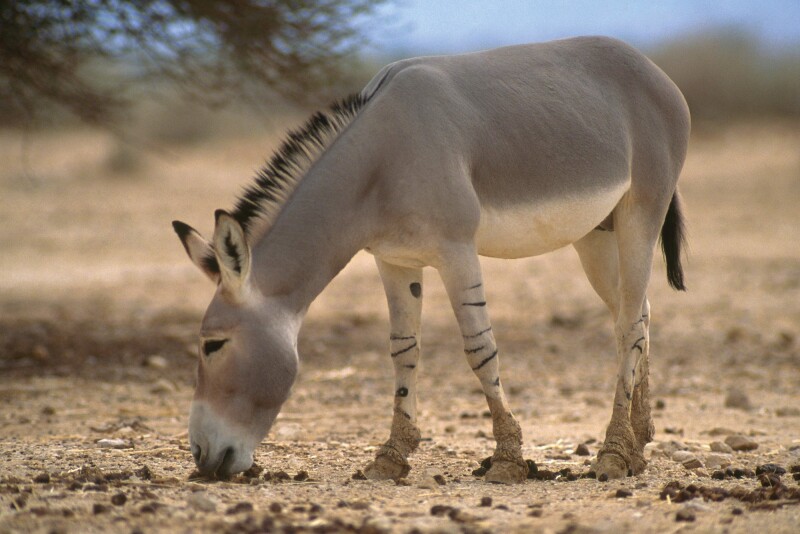
The habitat suitability of African wild ass (Equus africanus somaliensis) in Somaliland, eastern Africa. Models were fit with 61 presence points from interviews with agropastoralists. The respondents reported that African wild ass were either common (n = 2) or recently extirpated (n = 59) in their locality. Both maximum entropy and boosted regression tree models in conjunction with the interviews show that African wild asses are confined to about a third of the country. Additional information from the interviews suggests that the population is dangerously low or extirpated from Somaliland (Evangelista et al. 2018). Photograph: Mark D. Phillips, Science Photo Library.

Bottom-up programs that use CBM data to achieve specific management outcomes may help to establish or reinforce institutional or governance structures supportive of comanagement and monitoring evidence-based planning (e.g., Huntington 1992, Wilson et al. 2018). For example, the Yukon River Inter-Tribal Watershed Council's bottom-up Indigenous Observation Network has integrated top-down elements in terms of water quality research to align multiple interests and concerns in a large-scale setting spanning multiple tribal groups across the United States and Canada (Wilson et al. 2018). The combination of Somaliland goat herders’ information about presence of species, obtained via remote sensing and modeling, to derive species distribution estimates in eastern Africa (box 2, figure 3) further illustrates benefits obtained from a combination of top-down and bottom-up approaches.

**Box 2. Combining goat herders’ information with remote sensing and species distribution models to identify potential wildlife ranges in Somaliland.**

Knowing the potential range of species is important for determining habitat requirements and conservation status and for informing decision-making. In many remote and inaccessible regions, information on wildlife is scant or does not exist. A solution in such regions may be to connect knowledge of local resource users with remote sensing of vegetation and species distribution models. In 2016 and 2017, Evangelista and colleagues (2018) conducted 195 interviews with agropastoral men and women in Somaliland near the Horn of Africa and collected presence and absence information for 38 species of wildlife through interviews with goat herders who were shown photographs of relevant species. Remote sensing data on environmental variables and the information on where species have been recorded were used to draw maps showing the distribution of similar environments (based on 12 environmental predictor variables), thereby predicting the potential distribution of each species. The information was used with two types of species distribution models (maximum entropy and boosted regression tree). The study demonstrated how knowledge of experienced resource users could be combined with remote sensing data and species distribution models to identify the potential range of wildlife in data poor regions. As an example, figure 3 shows the relative habitat suitability for African wild ass (*Equus africanus somaliensis*) in Somaliland.

**Figure:**
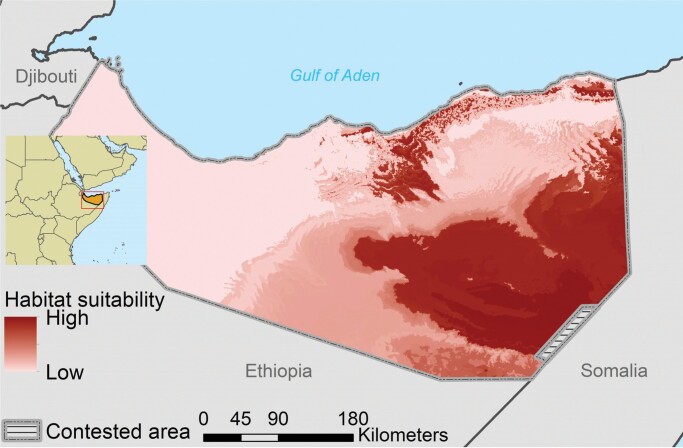

## Community-based environmental monitoring: Perspectives from the Arctic

Given the nature of changes in Arctic socioenvironmental systems requiring community-level response and considering the disproportionately large number of CBM programs in the region (table 1), we examined characteristics of Arctic CBM programs in more depth. Previously, as a task under the Sustaining Arctic Observing Networks (SAON) initiative, Johnson and colleagues (2016) had catalogued 81 Arctic CBM programs. Building on this work and an expanded literature review, Danielsen and colleagues (2020) identified a total of 170 CBM programs across the Arctic. These authors chose a subset of 45 programs for an in-depth analysis to reflect the widest possible set of situations and issues, including breadth in geographical coverage and attributes being monitored. They then sent each of these CBM programs a 50-question survey, which was completed by 30 programs (67% survey response rate; see the supplemental material). CBM practitioners (one respondent for each program) were asked general questions relevant to all Arctic monitoring systems, as well as questions of particular relevance to CBM programs. The report on this work (Danielsen et al. 2020) describes the characteristics and coverage of the CBM programs and identifies the format of resulting knowledge products. For each program, the report assessed its ability to contribute, or probably contribute, to better-informed decisions and better-informed processes in key economic sectors in the Arctic, which CBM programs could—or probably could—contribute to achieving the objectives of 10 multilateral agreements in the Arctic, and which of the 17 UN Sustainable Development Goals (SDG) the CBM programs could contribute to achieving. All CBM programs were assessed by one coauthor; in the few cases in which there was doubt in the evaluation of relevance to policy or decision-making, consensus was reached through discussion with other coauthors. Drawing on survey respondents, workshops were held in Greenland, the Komi and Sakha Republics, Québec, and Alaska (Fidel et al. 2017, Johnson et al. 2018, Enghoff et al. 2019) with CBM practitioners and community members who provided additional information on CBM practices and challenges (Danielsen et al. 2020).

**Figure 4.**
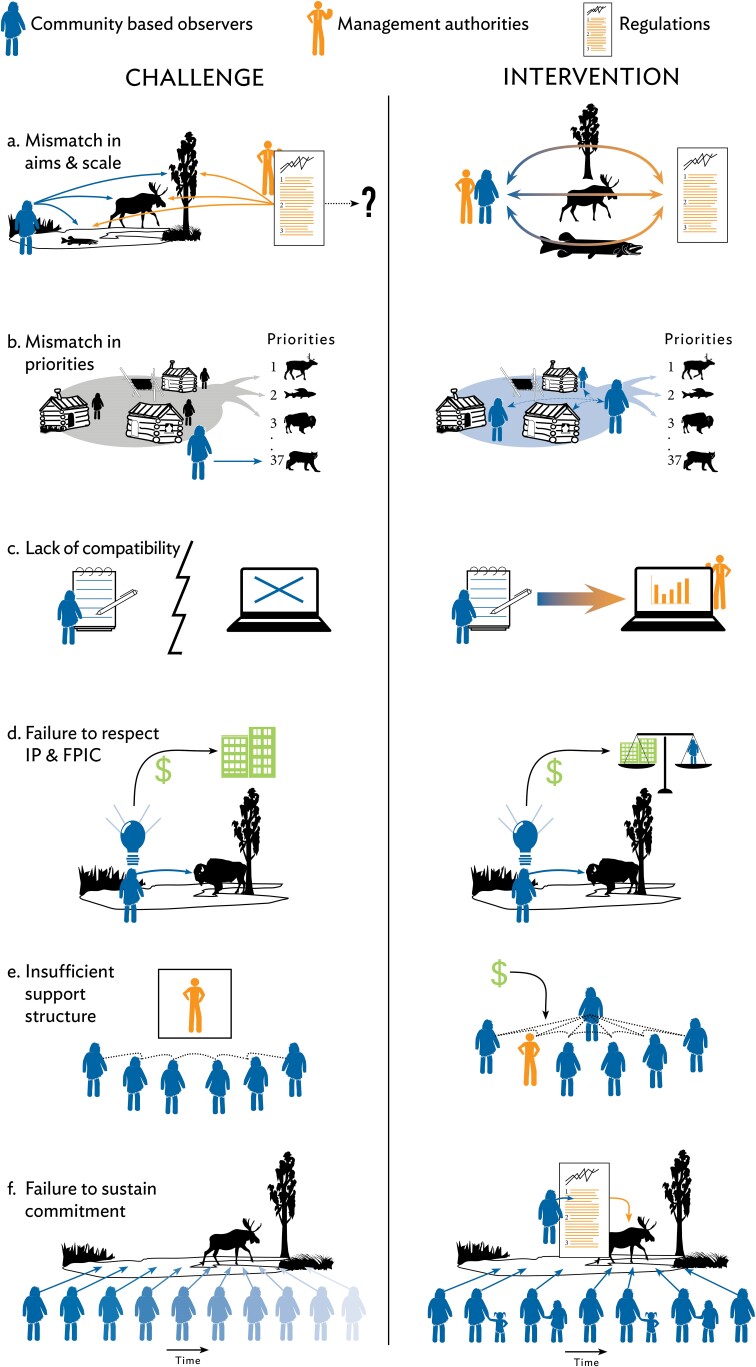
Summary of challenges and interventions in linking bottom-up and top-down observing. Each panel corresponds to issues and interventions discussed in the text. Input through community observations into resource management regulations is shown in orange, and transfer of intellectual property, symbolized by light bulb in panel (d), into applications and associated generation of revenue (in US dollars) are shown in green. The most promising interventions include a focus on knowledge coproduction principles covering the appropriate scales and priorities (a, b), data management responsive to CBM needs and capacities (c), respectful and appropriate use of CBM data (d), use of proper incentives and support partnerships (e), and intergenerational engagement to sustain efforts (f). Abbreviations: FPIC, free, prior, and informed consent; IP, intellectual property.

In the present article, we synthesize findings from this past report, combining them with a review of the global CBM literature completed for this publication and integrating findings from other case studies (such as those shown in boxes 2–4), to examine the benefits, challenges, and possible interventions to better connect top-down and bottom-up approaches in environmental monitoring. The analysis (table 1) shows a roughly equal proportion of bottom-up and top-down approaches, diverging somewhat from the key finding of a preponderance of bottom-up Arctic programs in our global CBM review (table 1). The survey indicated that the majority of CBM programs mostly informed decisions at the village or regional (subnational) level (73 and 66% of all surveyed)—a key aspect of bottom-up, community-driven efforts that can help address resource management or climate change adaptation challenges and bridge scales (Armitage et al. 2011, Danielsen et al. 2021 [this issue]). Nevertheless, many of the programs informed decisions at the national (40%) and international level (13%). The latter are of particular interest in exploring good practices to link top-down and bottom-up efforts. The Finnish Meteorological Institute's monitoring of snow depth since 1909 (Leppänen et al. 2016) and the recently launched Community Snow Observations (CSO) effort (Hill et al. 2018) are examples of programs with potential to tie into global climate-scale observing programs, specifically the World Meteorological Organization's Global Cryosphere Watch (Key et al. 2015). CSO, a citizen-science program that combines bottom-up and top-down attributes as it builds on interests of the winter backcountry recreation community, also illustrates the value of better links between community-driven and scientist-led programs. In this case, the link to the scientific research community provided funding and access to satellite data that extends the geographic reach and information value of individual snow measurements (Hill et al. 2018).

**Figure 5.**
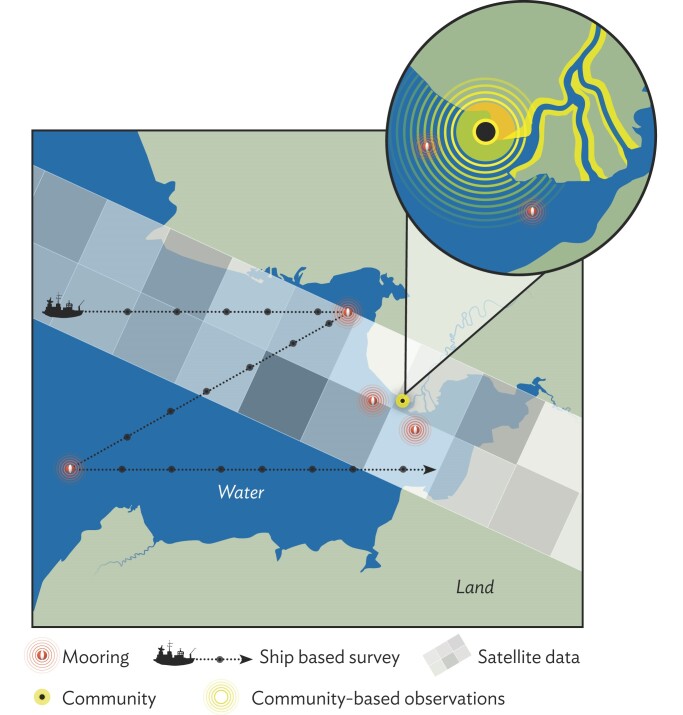
Schematic illustrating the potential for mismatched observational scales in relation to areas of interest to community members—for example, for resource harvests (the yellow circles and corridors indicate the range over which such activities and associated observations extend, with greater distances covered along the river, including seasonal camps located further upstream).

**Box 3. Linking remote sensing and CBM to track and predict sea-ice conditions relevant to community ice use in Arctic Alaska.**

**Figure 6.**
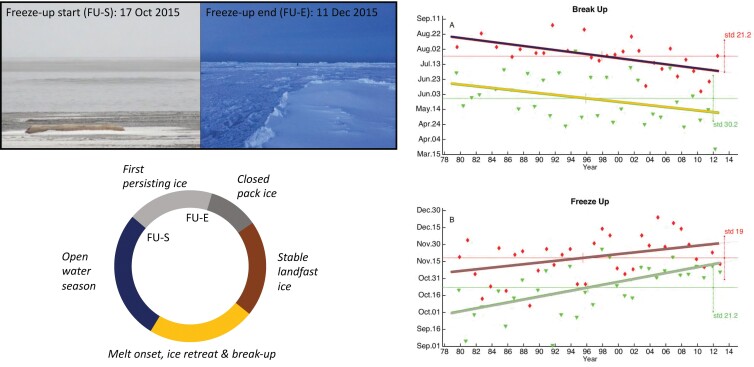
The seasonal ice cycle in northern coastal Alaska, showing the freeze-up season in grey and ice breakup season in orange (bottom left). Community observer Billy Adams’ photos illustrate start (open ocean shows first signs of persistent slush ice, Iñupiaq: qinu) and end of freeze-up season (new ice persists and is thick enough to walk on, Iñupiaq: sikuliaq). Satellite-derived time series of freeze-up and breakup start and end (trend lines for start and end of each correspond to colors in seasonal cycle at left), based on an algorithm developed from CBM, are shown on the right for 1979–2013 (Johnson and Eicken 2016). Definitions of start and end of freeze-up and breakup tied to important community activities, such as hunters prevented from running boats by persistent qinu or hunters able to pursue seals on sikuliaq, were linked to time series of ice concentration derived from satellite, to identify the timing of key events determining resource access over longer time periods.

Breakup and freeze-up of coastal sea ice determine timing and extent of several human activities, ranging from ice use by Indigenous hunters to coastal shipping (figure 6; Eicken et al. 2009, Stroeve and Notz 2018). However, while loss of Arctic sea ice has been well studied, changes in its seasonal cycle have received less attention. Moreover, different definitions of seasonal transitions (Johnson and Eicken 2016) and the coarse spatial resolution of satellite data as illustrated in figure 5 may affect the relevance of such data for community concerns. On the basis of community observations of sea ice use, Johnson and Eicken (2016) developed an algorithm to extract breakup and freeze-up timing from passive microwave satellite data. This effort drew on development of a database of ice use and relevant ice features, emerging from an unstructured, schema-less approach that provided greater flexibility for Inupiaq and Yupik sea-ice experts to record and share observations (Eicken et al. 2014). It was also informed by participatory scenario development in coastal communities to help identify key drivers, uncertainties, and high priority indicators (Preston and Lovecraft 2017). Data from 1979 to 2013 show the start of breakup arriving earlier by 5–9 days per decade and freeze-up start arriving later by 7–14 days per decade in the coastal seas of northern Alaska (figure 6). The trends toward a shorter ice season observed over the past several decades point toward a substantial change in the winter ice regime by mid-century with incipient overlap of the end of the freeze-up and start of the breakup season as defined by coastal ice users. Such information is relevant both in the context of understanding large-scale climate change (Stroeve and Notz 2018), as well as informing planning and decision-making at the local level. The timing of seasonal transitions such as ice freeze-up and breakup is a prime example of shared essential variables that benefit from both CBM and remote sensing observations and relate to issues such as food security that link different applications and concerns, as it was articulated by ICC (2015) from an Indigenous perspective and Global Cryosphere Watch (Key et al. 2015) from an international operational observing system perspective.

**Box 4. Aligning NASA research priorities, Indigenous knowledge, and local climate priorities.**

**Figure 7.**
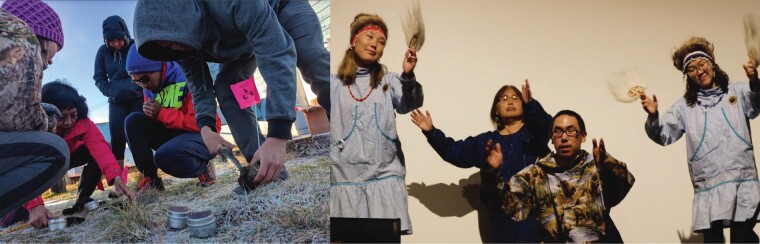
Kwethluk youth, community members, and University of Alaska Fairbanks scientists monitor soil moisture (left) to address local erosion issues accelerated by a warming climate, and performed a Yup'ik dance they created about their monitoring project (right) locally and at the international GLOBE conference in Killarney, Ireland.

Aligning top-down observing efforts with local needs and priorities for environmental observing is challenging, but not insurmountable. The Arctic and Earth Science, Technology, Engineering, and Math (STEM) Integrating Global Learning and Observations to Benefit the Environment (GLOBE) and NASA Assets (SIGNs) project uses a cocreated citizen science approach to craft local monitoring efforts that are motivated by input from elders and community leaders to address a pressing climate-related data need (Spellman et al. 2018). University of Alaska Fairbanks and NASA scientists collaborate with educators, youth, and long-term community members from Alaskan communities to codesign a monitoring project that addresses the local need. They work to align the effort with a NASA research priority using international monitoring protocols from the GLOBE program, an active 25-year-old program that serves as one of NASA's ground-truthing programs operating in over 120 countries. For the community of Kwethluk, Alaska, a team cocreated a soil moisture and erosion monitoring program that addressed the rapid erosion and loss of homes into their river related to permafrost thaw (figure 7). Youth involved in the project used their data to advocate for local erosion policy action, while NASA used their GLOBE soil moisture data for their Soil Moisture Active–Passive satellite mission. Youth used their new knowledge to create a Yup'ik dance on their project in collaboration with local elders and knowledge holders to communicate their results to their community and to the GLOBE and NASA international community (figure 7).

How well developed are links between the Arctic CBM programs surveyed and global frameworks? Arctic governments (with a few exceptions) have adopted ten international environmental agreements (Danielsen et al. 2020). Linking these environmental agreements to on-the-ground decision-making is a major challenge, however. We found that Arctic CBM programs could contribute to achieving the objectives of all ten agreements of particular relevance to the Arctic (Danielsen et al. 2020). Two agreements stand out as being particularly suitable for such contributions: The United Nations Framework Convention on Climate Change (UNFCCC) and the Convention on Biodiversity. A total of 100% and 80%, respectively, of the CBM programs evaluated could contribute to achieving the objectives of these agreements, such as by increasing community resilience. Our findings suggest that Arctic CBM programs may become important avenues for the implementation of environmental agreements in the Arctic, motivated—for example, by enhanced local-scale responses to rapid change.

Separate from the ten agreements, the UN SDG framework, adopted in 2015, has emerged as a key driver to enhance sustained observing and response at the global scale (Steffen et al. 2018). The SDGs are particularly relevant in helping to advance and align concerted action in regions of concern, such as the Arctic, low-lying island nations, and other areas under near-term threat (Mechler et al. 2019). We compared the CBM questionnaire responses with the 169 SDG targets for the 17 SDG goals and found that Arctic CBM programs contribute to achieving 16 of the 17 SDGs in the Arctic. Through improved data sharing and the wider use of digital platforms and global data repositories, CBM programs also could contribute to the seventeenth goal on technology partnerships (Johnson et al. 2021 [this issue]). These contributions also were echoed by our assessment of links between CBMs and economic sectors in the region. We found that CBM efforts contributed to better-informed decisions or better-documented processes in key economic sectors in the Arctic region: hunting or herding (60% of the CBM programs, *n* = 30), forestry (47%), fisheries (40%), shipping (37%), tourism (37%), and mineral and hydrocarbon extraction (20%).

## The role of Indigenous and local knowledge

The Inuit Circumpolar Council (ICC 2016) defines Indigenous knowledge as “a systematic way of thinking applied to phenomena across biological, physical, cultural and spiritual systems. It includes insights based on evidence acquired through direct and long-term experiences and extensive and multigenerational observations, lessons and skills.” Indigenous knowledge is embedded in a worldview, with a focus on practice, adaptation, and innovation that is directly tied to community value systems and decision-­making. Local knowledge—although it is not necessarily tied to broader community value systems—is also oriented toward a response and decision-making (Eicken 2010, ICC 2016, Magni 2017; see also box 1). The global-scale review of CBM programs indicates that Indigenous or local knowledge perspectives are part of roughly 15% of all CBM programs identified. In the Arctic, one third of programs are linked to Indigenous or local knowledge (table 1). This proportion is even higher in the Arctic survey results, where self-reporting identified Indigenous or local knowledge as being part of roughly half of all programs.

Because of their cross-disciplinary, holistic, empirical attributes and with their emphasis on management outcomes desired by individuals, communities, institutions, and organizations, Indigenous and local knowledge can help to bridge bottom-up and top-down monitoring and observing approaches (figure 1). This bridging function extends from guiding observations into variables and scales most relevant to implementation outcomes, to ensuring that observing system data and information obtained from climate- and global-scale programs support local-scale action. The processes in place for large-scale observing system design, such as the GOOS Framework on Ocean Observations (Lindstrom et al. 2012), value-tree analysis (STPI and SAON 2017), or biodiversity and ecosystem monitoring systems (CAFF 2013) typically operate several steps removed from community-level concerns and priorities, as is illustrated by the sea ice climate data variables shown in figure 2. These variables, typically collected at a coarse scale, are not as relevant to coastal community concerns as local-scale composite variables such as ice stability or trafficability that emerged as key priorities out of CBM efforts (figure 2). More broadly, studies have shown that incorporating Indigenous or local knowledge in CBM broadens perspectives and enhances outcomes (Alessa et al. 2016, Tengö et al. 2021 [this issue]). Finally, Indigenous and local knowledge can provide important evidence and context in establishing loss and damage as a result of climate change. Within the UNFCCC, such evidence that ties community impacts and change to climate-scale data obtained from earth system models and top-down observing programs can help to establish causal relationships and inform compensation for loss and damage suffered (Huggel et al. 2015). Below, we will examine in more detail how the bridging function of Indigenous and local knowledge can best be realized in observing system and CBM program design.

## Connecting top-down and bottom-up approaches: Benefits and challenges

Drawing on the three principal sources of information for this study detailed above (global literature review, review of Arctic CBM literature under SAON, and analysis of self-reported survey; see also the supplemental material), we identify six broader aims, associated benefits, and challenges that derive from improved links between top-down and bottom-up observing approaches. Challenges that may prevent these benefits from being realized also are discussed and summarized in figure 4.

### Improved fit between aims and scales in monitoring program

Linking top-down and bottom-up efforts can help to achieve better fit across different observational scales. CBM typically focuses on phenomena and processes at a fine scale commensurate with management or mitigating actions. In contrast, top-down, global scale observing systems address climate or ecosystem scale variables that matter to local communities but may not be as relevant if collected at a coarse scale and with insufficient granularity (illustrated for coastal and marine systems in figures 2 and 5). Such potential mismatch can be addressed through downscaling and upscaling of observations at the planning and implementation stage (Pratihast et al. 2016), including CBM-derived guidance on placement of sensor systems that are part of larger-scale top-down efforts (box 3, figures 5 and 6). Both the alignment of aims and the integration of remote sensing and *in situ* observations are further advanced through careful selection of target monitoring or observing variables that serve a larger constituency and provide shared benefits (figure 2).

Nevertheless, mismatches in aims and missions of government agencies and local entities continue to hamper ability or interest of management agencies to access, understand, and act on community-driven observations and guidance (figure 4a; Eicken 2010, Johnson et al. 2015, 2016, 2018, Lubilo and Hebinck 2019). Despite recent progress (Armitage et al. 2011, Kendall et al. 2017, Tengö et al. 2017), government agencies and academia continue to struggle to understand the nature and relevance of CBM and the Indigenous and local knowledge that informs many CBM efforts (table 1). Misconceptions include a perceived lack of CBM reliability and failure to appreciate equivalency of information generated through CBM and by professional scientists (Johnson et al. 2015, Costa et al. 2018). In part, historical and power relationships may create an adversarial dynamic—for example, between multilevel actors that are part of comanagement or between researchers and community members (Armitage et al. 2011, Long et al. 2016). Bureaucratic or political hurdles and lack of resources may make it difficult for government agencies to rely on CBM for decision support. The lack of reward structures in academia for work focused on actionable, solutions-oriented science remains problematic as well. Finally, international bodies advising governments on resource management are slow to establish procedures that take CBM observations and knowledge into account (Nordic Council of Ministers 2015, Danielsen et al. 2017, PAME 2017).

### Better match between observing program and community ­priorities

Many of the benefits that derive from CBM and well aligned top-down observing, such as filling critical information needs for local decision-making or ensuring sustainability of relevant programs, can be tied to well aligned priorities between communities and observing programs. When priorities align, as in the Arctic and Earth SIGNs project (box 4), substantial benefits can be achieved, such as local learning and action alongside robust international data sets.

Contrasting priorities between what is designated to be observed and what is valued by communities present a challenge at different levels (figure 4b). Although it is locale dependent, many communities value individual and community health, food security, economic opportunities, and other aspects of fate control, such as participation in the regulatory process or place-based education. In contrast, many observing programs focus on topics on the basis of outside perspectives, some directly derived from top-down, large-scale frameworks, and may address community priorities only marginally or not at all. University researchers often focus on large-scale processes that may be of little interest at the local level (figure 5). Regulatory frameworks may constrain government agencies on the type and scales of information that is collected. Communities are diverse, and establishing monitoring priorities that reflect consensus can be difficult (Wheeler et al. 2016).

### Greater compatibility between observing methodology and data management

Codesign and cocreation of observing and data management protocols (Shirk et al. 2012) is an effective mechanism to overcome the major interoperability challenges that hamper integration of observing systems (Parsons 2013, Godøy and Saadatnejad 2017). The same holds true in principle for linking CBM programs to large-scale, top-down efforts but is poorly explored in practice (Pulsifer et al. 2012, Fidel et al. 2017, Johnson et al. 2021 [this issue]). Such interoperability challenges can be tied to the disconnect between scientists’ focus on tracking state variables and system dynamics and outcomes-oriented observing in community-driven monitoring (figures 1 and 4c; Pulsifer et al. 2011, 2014). The latter typically focus on a single topic, but often draw on a broad suite of tracked variables, many embedded in Indigenous and local knowledge (Krupnik et al. 2010). The former, in contrast, attempt to integrate data arising from multiple sources to inform systems-level understanding and predictive skills in a broader range of applications (Lindstrom et al. 2012). The example in figure 2 illustrates how observing requirements for different variables flow from multiple applications, which, in turn, define the underlying observing and data networks.

Mismatches in the scale and granularity of data generated and managed through these networks also play into interoperability challenges. Therefore, data derived from satellites, scientific transects, or point sources are associated with very different data format, entry, curation, and archival modalities compared with CBM data obtained across a broader landscape on the basis of resource use and other factors (figure 5). The latter type of data often are excluded from global-scale data management centers because of perceived incompatibility and concerns about intellectual property rights and licensing. At the same time, incorporation of CBM outputs and perspectives into research has been found to enhance the quality of the science (box 3, figure 6; Mercer et al. 2010, Eerkes-Medrano et al. 2017).

### Respect of Indigenous intellectual property rights and free, prior, and informed consent

Respecting the rights of participating Indigenous and local communities as central aspects of all CBM programs is critical to successful codesign and cocreation between top-down and bottom-up approaches. Best practices in collaborating with Indigenous and local communities have been formulated (e.g., Borrini et al. 2004, Tengö et al. 2021 [this issue]). Careful consideration of ethics and methodology of knowledge sharing can result in greater recognition of community priorities and concerns, with better information products and support through top-down observing efforts (Castleden et al. 2012).

CBM programs operate within a broader context of research practice in which communities often are approached by well-intentioned outsiders interested in collaboration but without long-term commitment to understanding the local context of knowledge production and use (David-Chavez and Gavin 2018). Past failures of research collaborations to deliver final products that meet community information needs have led to greater sensitivity to ethics in research practice. This includes the need for awareness of and respect for existing protocols and frameworks for meaningful engagement of Indigenous peoples on the basis of Indigenous rights, such as free, prior, and informed consent (FPIC). Some guidelines exist that describe how to appropriately engage Indigenous and local knowledge (e.g., the Tkarihwaié:ri Code; CBD 2011, see also Nickels et al. 2007). Indigenous communities and organizations have raised the need for regionally appropriate and specific ethics protocols and research agreements (ITK 2018) and examined FPIC through a northern lens (Gladstone and Singleton-Polster 2016). However, more work is needed to advance implementation of specific protocols—for example, at the level of Institutional Review Boards in Alaska.

Some research and CBM programs have unclear agreements on data ownership and use (Costa et al. 2018). It is important that communities maintain control over data and that community members have access to the data with long-term data storage solutions as part of CBM design (Johnson et al. 2021 [this issue]). CBM can be a very important step in the efforts of Indigenous and local communities to claim their rights to knowledge and their share of any benefits accruing from this knowledge through—for example, the access and benefit-sharing mechanism of the Convention on Biodiversity. This, however, requires adherence to FPIC and clear agreements on data ownership and data use that prevent potential misuse, such as private companies using CBM-derived information for their own commercial benefit without providing any compensation (Posey 1998).

### Sufficient organizational support structures

Community-driven CBM programs and activities that connect successfully with top-down approaches hinge on organizational support structures that sustain the effort from the community up to the government level. These include institutional buy-in, long-term employment or volunteers, and sustainable funding. When the local and larger scale support structures align, monitoring efforts benefit from long-term continuous observations and result in cost-effective, sustainable monitoring programs with strong local participation that are culturally relevant and have scientific value (Fry 2011).

However, such support structures are often lacking. Programs may be established without any insight into existing organizational or institutional landscapes in the area. Instead of properly incorporating CBM activities into local organizations with a track record of success, parallel island structures are set up that wither and detract from existing successes (Costa et al. 2018). Typically, natural resource management programs require 5–10 years of external support before self-sufficiency, with some CBM programs not sustained over this minimum period of time and therefore unable to achieve their main objectives.

CBM programs also may be initially developed with scientists in academic institutions that provide the organizational, administrative, or technological support but then need to transition these support roles to an appropriate community-run entity over the long term. Such research-to-operations transitions, although it is fundamental to the evolution of observing system implementation in general, remain challenging (Wilson 2010). This is true for both top-down and bottom-up approaches, as has been illustrated by recent reviews (Lee et al. 2019).

### Sustained commitment of community members

To fully capture the effects of natural variability in climate or environmental systems typically requires observations at the timescale of a decade and beyond (e.g., Eicken 2010). Sustaining community members’ commitment beyond this time scale accrues other benefits, such as greater effectiveness in translating monitoring results into management guidance. The strength of some—though not all—of the comanagement institutions in Arctic Alaska and the Inuvialuit Settlement Region with a long history of, for example, marine mammal CBM (Huntington 1992, Meek 2013, Ostertag et al. 2018) speaks to this issue.

Nevertheless, fatigue among community members and participant turnover at the community level were considered significant challenges for one in five of Arctic CBM programs surveyed (Danielsen et al. 2020). Such turnover potentially jeopardizes long-term CBM sustainability, including continuity of the resulting data records (Conrad and Hilchey 2011). Frequent staff turnover at the management authority level is problematic as well. Poor fit between CBM design and the local context is a key source of loss of engagement among community members (see above, figure 4f). This issue is exacerbated by observing protocols that consume too much time and resources, as well as insufficient feedback on CBM results and management outcomes. Also, proper recognition of CBM observers’ contributions and central role in achieving management outcomes is critical, including use of CBM information for actual management decisions at higher levels.

## Connecting top-down and bottom-up approaches: Interventions

To reap the full benefits from closer links between top-down and bottom-up observing approaches, challenges identified (figure 4) need to be overcome. Danielsen and colleagues (2020) specified 38 different interventions to address such challenges. We synthesize and expand these findings to arrive at broader conclusions and potential next steps.

A major factor in addressing challenges is to rely on codesign, comanagement, and coproduction principles. Although definitions of knowledge coproduction may vary (see box 1), observing and monitoring efforts benefit from pragmatic approaches that draw on some of the following. First, involve community representatives and CBM program facilitators in observing program planning and evaluation (figure 4a; Tredick et al. 2017). Protocols should prioritize community feedback and involvement (figure 4b). Participatory scenarios may help with prioritization (box 3, Preston and Lovecraft 2017). Consideration should be given to community data priorities and needs (box 4; Shirk et al. 2012). Second, further develop good practices and protocols to allow government agencies and international scientific organizations and management bodies to incorporate CBM-derived information in their decision-making (figure 4c). Third, focus on program sustainability in CBM design and implementation. Tie into the existing organizational and governance structures in the area and use data collection tools and approaches that are easily incorporated into daily community activities (figure 4e, 4f; Ison 2008, David-Chavez and Gavin 2018). Fourth, include youth and school groups to build future monitoring capacity and sustain interest across generations (figure 4f, box 4; Spellman et al. 2018). Fifth, encourage the use of protocols to enable respectful engagement with Indigenous and local knowledge (figure 4d).

Equitable support to team members from communities that is on par with that received by scientists is critical (salary, recognition as coauthors; figure 4e). Regular feedback to community members with CBM findings and updates on how the findings are used for decision-making are an important part of incentive structures. Recognition of scientist engagement also is important, including added emphasis on community engagement in academic and government assessment and promotion.

Data comanagement with an emphasis on data ownership and use rights that draws, for example, on concepts of Indigenous data management (Pulsifer et al. 2011), is an important corollary to the coproduction approaches outlined above. Encouraging managers of scientific data repositories to adjust data formats to become receptive to data from CBM programs and to provide focused support of CBM programs keen to connect with scientific data repositories are further steps to take.

Finally, a great help in overcoming challenges is raising awareness within government agencies and scientific organizations on the value of CBM, Indigenous and local knowledge, and the usefulness of incorporating information from CBM programs into scientific data repositories in support of systems-level understanding and future decision-making. Such work also may bring different constituencies together to share information, promote advocacy on the importance of using CBM-derived information, and provide training on CBM activities and evidence collection from CBM as part of a research and monitoring portfolio. In all of this, it needs to be recognized that institutionalizing CBM programs within existing organizations is a capacity building process that takes time and must be based on trust and confidence.

## Conclusions

Significant benefits for environmental management, planning, and decision-making can be derived from observing or monitoring activities that draw on both bottom-up and top-down approaches. An example of the former are community-driven efforts that contribute to outcomes desired by a local community, whereas the latter may be represented by global-scale observing systems that focus on tracking essential variables defined by research scientists to describe the state of a large-scale environmental system. However, these approaches may overlap both in scale and target variables. Therefore, significant benefits for a range of user groups can be derived by linking or combining methodologies associated with both approaches. The example of sea ice information needs at the intersection of different societal benefits and applications (figure 2, box 3) illustrates the necessity to align observing priorities, identify variables that provide shared benefits for different users, and capture data at the spatial and temporal resolution associated with particular applications.

To reap the full benefits from combining these two approaches, a number of challenges discussed in this study need to be overcome. Although the interventions presented above can help clear a path, progress along that path requires resources and action. From this study and related work, conclusions and recommendations for how to achieve progress emerge. Most importantly, the implementation of principles of codesign, comanagement, and coproduction is facilitated greatly by focusing on pressing societal problems at a scale that intersects interests of both local communities in a particular region and large-scale observing efforts. The latter, in particular as exemplified by satellite remote sensing programs, often have the resources and government support for sustained observations, but need to build and maintain partnerships to effectively link to local-scale concerns. The European Union's Copernicus program dedicated to monitoring and forecasting of key Earth subsystems, such as for the oceans (Le Traon et al. 2019), is an example of an effort commanding major financial resources and good links into the government sector that holds unrealized promise for CBM and local-scale information needs. Several of the activities highlighted in this study, including work summarized in boxes 2–4, can serve as examples of how to connect satellite remote sensing to community concerns.

Therefore, to make progress we see investments into regional-scale observing codesign efforts centered around a core, high-priority theme as critical to building sufficient momentum and connecting different resources for efforts to be meaningful and sustainable. Programmatically, in parallel with such investments, existing programs may need to merge or reinvent themselves to adapt in a rapidly evolving observing landscape. Furthermore, ensuring that all partners have the means to build capacity for engagement, communication, and coordination across a diverse set of jurisdictions and sectors is key. In the Arctic, the food security framework put forward by the Inuit Circumpolar Council–Alaska (ICC 2015) may serve to illustrate this point. Food insecurity and the quest for Indigenous food sovereignty have informed food security concepts within the Inuit worldview that transcend food availability and access, extending to, for example, the ability to preserve traditional food systems in ways that nourish and sustain the values underlying cultural identity (ICC 2015, 2020).

In the Arctic, climate change is a driver, but so are numerous other factors such as costs of harvesting resources. These factors are subsumed under six dimensions of Alaskan Inuit food security, including availability, Inuit culture, and decision-making power and management, and a total of 58 specific drivers of food security (or insecurity). The ICC's work in documenting food security from an Inuit perspective lays a foundation on which observing system codesign—supported through global or regional-scale networks and alliances—can advance through the process of defining shared essential variables to implementation of observing and data networks. Therefore, the ICC food security framework can inform collaboration at the interface between CBM and global-scale satellite observing programs (figure 2), leading to integrated observing plans (figure 5). If similar processes of documenting Indigenous understandings of food security were replicated by other Arctic Indigenous peoples, the integration would become more robust and reflective of the diversity of knowledge systems in the Arctic. Essential for progress along this path is the equitable support of Indigenous and local community experts and representatives, and the capacity of large-scale observing program partners to meaningfully engage at a scale and in a region of interest to both (Pulsifer et al. 2011, David-Chavez and Gavin 2018). As coproduction is an ongoing process, data management and data networks need to provide information products of value to all partners and data users.

## Supplementary Material

biab018_Supplemental_Files

## Author Biographical

Hajo Eicken (heicken@alaska.edu) is a professor and the director of the International Arctic Research Center, University of Alaska Fairbanks, in Fairbanks, Alaska, in the United States. Finn Danielsen and Michael K. Poulsen are ecologists and Martin Enghoff is a social scientist at the Nordic Foundation for Development and Ecology in Copenhagen, Denmark. Josephine-Mary Sam is a postdoctoral researcher at the International Arctic Research Center, at the University of Alaska Fairbanks, in Fairbanks, Alaska, in the United States. Maryann Fidel is an environmental scientist at the Yukon River Inter-Tribal Watershed Council, in Anchorage, Alaska, in the United States. Noor Johnson is a research scientist at the National Snow and Ice Data Center, at the University of Colorado, Boulder, in Boulder, Colorado, in the United States. Peter Pulsifer is an associate professor and the associate director of the Geomatics and Cartographic Research Centre at Carleton University, in Ottawa, Ontario, Canada. Lisbeth Iversen is an adjunct researcher at the Nansen Environmental and Remote Sensing Center, in Bergen, Norway. Olivia A. Lee and Katie Villano Spellman are research assistant professors at the International Arctic Research Center, at the University of Alaska Fairbanks, in Fairbanks, Alaska, in the United States.
