# Supplementary material for: Connecting Top-Down and Bottom-Up Approaches in Environmental Observing
Source: Bioscience. 2021 Apr 28;71(5):467–83. doi: 10.1093/biosci/biab018 (PMC8106998; doi:10.1093/biosci/biab018)
Supplement: biab018_Supplemental_Files [file biab018_supplemental_files.zip › AuthorshipStatement.docx]

*Department in BioScience. Special Section on Monitoring.*

**Connecting Top-Down and Bottom-Up Approaches in Environmental Observing**

Hajo Eicken^1^, Finn Danielsen^2^, Josephine-Mary Sam^1^, Maryann Fidel^3^, Noor Johnson^4^, Michael K. Poulsen^2^, Olivia A. Lee^1^, Katie Villano Spellman^1^, Lisbeth Iversen^5^, Peter Pulsifer^6^, Martin Enghoff^2^

**Authorship Statement – Contributions by each author**

Global literature review: JMS, HE

Arctic literature review: NJ, MF, FD, PP

Arctic CBM survey: MF, NJ, FD

Workshop organization and products: FD, MF, NJ, PP, OAL, KVS, LI, HE

Article concept development: HE, FD

Figure development: HE, FD, MKP, KVS

Article writing: HE, FD, JMS, MF, NJ, MKP, OAL, KVS, LI, PP, ME
