# Supplementary material for: Connecting Top-Down and Bottom-Up Approaches in Environmental Observing
Source: Bioscience. 2021 Apr 28;71(5):467–83. doi: 10.1093/biosci/biab018 (PMC8106998; doi:10.1093/biosci/biab018)
Supplement: biab018_Supplemental_Files [file biab018_supplemental_files.zip › Eicken_BioScience_Supplement_124ReviewedReferences.pdf]

Reference list of publications identified through review of relevant entries in Elsevier Scopus curated abstract and citation database

1. Aceves-Bueno E, et al. 2015. Citizen Science as an approach for overcoming insufficient monitoring and inadequate stakeholder buy-in in adaptive management: Criteria and evidence. Ecosystems 18:493-506.
2. Adams GL, Jennings S, Reuman DC. 2017. Community management indicators can conflate divergent phenomena: two challenges and a decomposition-based solution. Journal of Applied Ecology 54:883-893.
3. Alessa L, Kliskey A, Gamble J, Fidel M, Beaujean G, Gosz J. 2016. The role of Indigenous science and local knowledge in integrated observing systems: moving toward adaptive capacity indices and early warning systems. Sustainability Science 11:91-102.
4. Andow DA, Borgida E, Hurley TM, Williams AL. 2016. Recruitment and Retention of Volunteers in a Citizen Science Network to Detect Invasive Species on Private Lands. Environmental Management 58:606-618.
5. Augar N, Fluker M. 2014. Developing social media for community based environmental monitoring: i6doc.com.
6. Bandara RMS, Bhasin RK, Kjekstad O, Arambepola NMSI. 2013. Examples of cost effective practices for landslide monitoring for early warning in developing countries of Asia. Pages 581-588.
7. Becker CD, Agreda A, Astudillo E, Costantino M, Torres P. 2005. Community-based monitoring of fog capture and biodiversity at Loma Alta, Ecuador enhance social capital and institutional cooperation. Biodiversity and Conservation 14:2695-2707.
8. Bellfield H, Sabogal D, Goodman L, Leggett M. 2015. Case study report: Community-based monitoring systems for REDD+ in Guyana. Forests 6:133-156.
9. Biradavolu MR, Blankenship KM, George A, Dhungana N. 2015. Unintended consequences of community-based monitoring systems: Lessons from an HIV prevention intervention for sex workers in South India. World Development 67:1-10.
10. Bliss J, Aplet G, Hartzell C, Harwood P, Jahnige P, Kittredge D, Lewandowski S, Soscia ML. 2001. Community-based ecosystem monitoring. Journal of Sustainable Forestry 12:143-167.
11. Boutsis I, Kalogeraki V. 2013. Mobile stream sampling under time constraints. Pages 227-236.
12. Brammer JR, et al. 2016. The role of digital data entry in participatory environmental monitoring. Conservation Biology 30:1277-1287.
13. Brites AD, Morsello C. 2018. Effects of economic dependence and cooperative behavior over participation in monitoring the impacts of natural resource trade. Ecological Economics 147:365-372.
14. Brofeldt S, et al. 2014. Community monitoring of carbon stocks for REDD+: Does accuracy and cost change over time? Forests 5:1834-1854.
15. Brook RK, Kutz SJ, Veitch AM, Popko RA, Elkin BT, Guthrie G. 2009. Fostering community-based wildlife health monitoring and research in the Canadian North. EcoHealth 6:266-278.
16. Burgos A, Páez R, Carmona E, Rivas H. 2013. A systems approach to modeling Community-Based Environmental Monitoring: A case of participatory water quality monitoring in rural Mexico. Environmental Monitoring and Assessment 185:10297-10316.

17. Cappa F, Laut J, Nov O, Giustiniano L, Porfiri M. 2016. Activating social strategies: Face-to-face interaction in technology-mediated citizen science. Journal of Environmental Management 182:374-384.
18. Carlson T, Cohen A. 2018. Linking community-based monitoring to water policy: Perceptions of citizen scientists. Journal of Environmental Management 219:168-177.
19. Chandler M, et al. 2017. Contribution of citizen science towards international biodiversity monitoring. Biological Conservation 213:280-294.
20. Chapman S, Sullivan C, Palm C, Huynh U, Diru W, Masira J. 2016. Monitoring and evaluation to support adaptive co-management: Lessons learned from the Millennium Villages Project. Journal of Environmental Management 183:142-151.
21. Cliche L, Freeman L. 2017. Applying integrated watershed management in Nova Scotia: a community-based perspective from the Clean Annapolis River Project. International Journal of Water Resources Development 33:441-457.
22. Commodore A, Wilson S, Muhammad O, Svendsen E, Pearce J. 2017. Community-based participatory research for the study of air pollution: a review of motivations, approaches, and outcomes. Environmental Monitoring and Assessment 189 (art. 378).
23. Conrad C. 2006. Towards meaningful community-based ecological monitoring in Nova Scotia: Where are we versus where we would like to be. Environments 34:25-36.
24. Conrad CC, Hilchey KG. 2011. A review of citizen science and community-based environmental monitoring: Issues and opportunities. Environmental Monitoring and Assessment 176:273-291.
25. Conrad CT, Daoust T. 2008. Community-based monitoring frameworks: Increasing the effectiveness of environmental stewardship. Environmental Management 41:358-366.
26. Crall AW, Jarnevich CS, Young NE, Panke BJ, Renz M, Stohlgren TJ. 2015. Citizen science contributes to our knowledge of invasive plant species distributions. Biological Invasions 17 (art. A016):2415-2427.
27. Crall AW, Renz M, Panke B, Newman GJ. 2011. Is there a role for the public in monitoring invasive species? CAB Reviews: Perspectives in Agriculture, Veterinary Science, Nutrition and Natural Resources 6 (art. 23):1-7.
28. Cunha DGF, Marques JF, de Resende JC, de Falco PB, de Souza CM, Loiselle SA. 2017. Citizen science participation in research in the environmental sciences: Key factors related to projects' success and longevity. Anais da Academia Brasileira de Ciencias 89:2229-2245.
29. Danielsen F, Burgess ND, Balmford A. 2005. Monitoring matters: Examining the potential of locally-based approaches. Biodiversity and Conservation 14:2507-2542.
30. Danielsen F, Jensen AE, Alviola PA, Balete DS, Mendoza M, Tagtag A, Custodio C, Enghoff M. 2005. Does monitoring matter? A quantitative assessment of management decisions from locally-based monitoring of protected areas. Biodiversity and Conservation 14:2633-2652.
31. Danielsen F, et al. 2011. At the heart of REDD+: A role for local people in monitoring forests? Conservation Letters 4:158-167.
32. Dantas Brites A, Morsello C. 2017. Beliefs about the Potential Impacts of Exploiting Non-Timber Forest Products Predict Voluntary Participation in Monitoring. Environmental Management 59:898-911.
33. Deutsch WG, Ruiz-Córdova SS. 2015. Trends, challenges, and responses of a 20-year, volunteer water monitoring program in Alabama. Ecology and Society 20 (art. 14).

34. Dewachter S, Holvoet N. 2017. Intersecting social-capital and perceived-efficacy perspectives to explain underperformance in community-based monitoring. Evaluation 23:339-357.
35. Driscoll DL, Mitchell E, Barker R, Johnston JM, Renes S. 2016. Assessing the health effects of climate change in Alaska with community-based surveillance. Climatic Change 137:455-466.
36. Duvert C, Gratiot N, Némery J, Burgos A, Navratil O. 2011. Sub-daily variability of suspended sediment fluxes in small mountainous catchments - Implications for community-based river monitoring. Hydrology and Earth System Sciences 15:703-713.
37. Espino AN, Jr., Cinense MM, Salvador NC, Alberto AMP, Bauan RC. 2015. Community-based watershed monitoring system for biodiversity conservation in Lagmay AMFA, ed: Asian Association on Remote Sensing.
38. Farhan Ferrari M, de Jong C, Belohrad VS. 2015. Community-based monitoring and information systems (CBMIS) in the context of the Convention on Biological Diversity (CBD). Biodiversity 16:57-67.
39. Fernandez-Gimenez ME, Ballard HL, Sturtevant VE. 2008. Adaptive management and social learning in collaborative and community-based monitoring: A study of five community-based forestry organizations in the western USA. Ecology and Society 13 (art. 4).
40. Flores-Díaz AC, Chacón AQ, Bistrain RP, Ramírez MI, Larrazábal A. 2018. Community-based monitoring in response to local concerns: Creating usable knowledge for water management in rural land. Water (Switzerland) 10 (art. 542).
41. Garcia CA, Lescuyer G. 2008. Monitoring, indicators and community based forest management in the tropics: Pretexts or red herrings? Biodiversity and Conservation 17:1303-1317.
42. Garda C, Castleden H, Conrad C. 2017. Monitoring, restoration, and source water protection: Canadian community-based environmental organizations' efforts towards improving aquatic ecosystem health. Water (Switzerland) 9 (art. 212).
43. Gérin-Lajoie J, et al. 2018. IMALIRIJIIT: A community-based environmental monitoring program in the George River Watershed, Nunavik,Canada. Ecoscience 25:381-399.
44. Gillett DJ, Pondella Ii DJ, Freiwald J, Schiff KC, Caselle JE, Shuman C, Weisberg SB. 2012. Comparing volunteer and professionally collected monitoring data from the rocky subtidal reefs of southern California, USA. Environmental Monitoring and Assessment 184:3239-3257.
45. Haggitt TR, Mead ST. 2015. Makara estuary monitoring: Effects-based monitoring within a degraded, yet dynamic, coastal environment. Effects-based. Pages 364-370: Australian Coasts and Ports.
46. Hassenforder E, Ducrot R, Ferrand N, Barreteau O, Anne Daniell K, Pittock J. 2016. Four challenges in selecting and implementing methods to monitor and evaluate participatory processes: Example from the Rwenzori region, Uganda. Journal of Environmental Management 180:504-516.
47. Heistermann M, Jacobi S, Pfaff T. 2013. Technical Note: An open source library for processing weather radar data (wradlib). Hydrology and Earth System Sciences 17:863-871.

48. Henri DA, Jean-Gagnon F, Gilchrist HG. 2018. Using inuit traditional ecological knowledge for detecting and monitoring avian cholera among common eiders in the eastern Canadian Arctic. Ecology and Society 23 (art. 22).
49. Hockley NJ, Jones JPG, Andriahajaina FB, Manica A, Ranambitsoa EH, Randriamboahary JA. 2005. When should communities and conservationists monitor exploited resources? Biodiversity and Conservation 14:2795-2806.
50. Holck MH. 2008. Participatory forest monitoring: An assessment of the accuracy of simple cost-effective methods. Biodiversity and Conservation 17:2023-2036.
51. Humber F, Godley BJ, Ramahery V, Broderick AC. 2011. Using community members to assess artisanal fisheries: The marine turtle fishery in Madagascar. Animal Conservation 14:175-185.
52. Ishihara S, Boyles RM, Matsubayashi H, Del Barrio AN, Cebrian MR, Ishida A, Lapitan RM, Atabay EP, Cruz LC, Kanai Y. 2015. Long-term community-based monitoring of tamaraw Bubalus mindorensis on Mindoro Island. ORYX 49:352-359.
53. Jafari H, Li X, Qian L, Chen Y. 2015. Community based sensing: A test bed for environment air quality monitoring using smartphone paired sensors. Pages 12-17: Institute of Electrical and Electronics Engineers Inc.
54. Johnson N, et al. 2015. The contributions of community-based monitoring and traditional knowledge to Arctic observing networks: Reflections on the state of the field. Arctic 68:28-40.
55. Kendrick A. 2013. Canadian Inuit sustainable use and management of Arctic species. International Journal of Environmental Studies 70:414-428.
56. Kondo MC, Mizes C, Lee J, McGady-Saier J, O'Malley L, Diliberto A, Burstyn I. 2014. Towards participatory air pollution exposure assessment in a goods movement community. Progress in Community Health Partnerships: Research, Education, and Action 8:291-304.
57. Koss RS, Miller K, Wescott G, Bellgrove A, Boxshall A, McBurnie J, Bunce A, Gilmour P, Ierodiaconou D. 2009. An evaluation of Sea Search as a citizen science programme in Marine Protected Areas. Pacific Conservation Biology 15:116-127.
58. Kouril D, Furgal C, Whillans T. 2016. Trends and key elements in community-based monitoring: A systematic review of the literature with an emphasis on Arctic and Subarctic regions. Environmental Reviews 24:151-163.
59. Kumar N, et al. 2016. Environmental PCBs in Guánica Bay, Puerto Rico: implications for community health. Environmental Science and Pollution Research 23:2003-2013.
60. Lee DE, Bond ML. 2018. Quantifying the ecological success of a community-based wildlife conservation area in Tanzania. Journal of Mammalogy 99:459-464.
61. Lefland AB, Huff ES, Donahue B. 2018. A community forestry model linking research, management, education, and stakeholder engagement: Case study results from the Town of Weston, Massachusetts, USA. Small-scale Forestry 17:191-210.
62. Little KE, Hayashi M, Liang S. 2016. Community-Based Groundwater Monitoring Network Using a Citizen-Science Approach. Groundwater 54:317-324.
63. Lynch J, Eilam E, Fluker M, Augar N. 2017. Community-based environmental monitoring goes to school: translations, detours and escapes. Environmental Education Research 23:708-721.

64. Lyver POB, Timoti P, Jones CJ, Richardson SJ, Tahi BL, Greenhalgh S. 2017. An indigenous community-based monitoring system for assessing forest health in New Zealand. Biodiversity and Conservation 26:3183-3212.
65. Malakar Y. 2014. Community-based rainfall observation for landslide monitoring in western Nepal. Pages 757-763. Landslide Science for a Safer Geoenvironment: Volume 2: Methods of Landslide Studies, Springer International Publishing.
66. Mantyka-Pringle CS, et al. 2017. Bridging science and traditional knowledge to assess cumulative impacts of stressors on ecosystem health. Environment International 102:125-137.
67. McKay AJ, Johnson CJ. 2017. Identifying effective and sustainable measures for community-based environmental monitoring. Environmental Management 60:484-495.
68. Mesterton-Gibbons M, Milner-Gulland EJ. 1998. On the strategic stability of monitoring: Implications for cooperative wildlife management programmes in Africa. Proceedings of the Royal Society B: Biological Sciences 265:1237-1244.
69. Millar EE, Hazell EC, Melles SJ. 2018. The ‘cottage effect’ in citizen science? Spatial bias in aquatic monitoring programs. International Journal of Geographical Information Science:1-21.
70. Minoi JL, Yeo AW. 2014. Remote health monitoring system in a rural population: Challenges and opportunities. Pages 895-900: Institute of Electrical and Electronics Engineers Inc.
71. Molina E, Carella L, Pacheco A, Cruces G, Gasparini L. 2017. Community monitoring interventions to curb corruption and increase access and quality in service delivery: a systematic review. Journal of Development Effectiveness 9:462-499.
72. Monk J, Ierodiaconou D, Bellgrove A, Laurenson L. 2008. Using community-based monitoring with GIS to create habitat maps for a marine protected area in Australia. Journal of the Marine Biological Association of the United Kingdom 88:865-871.
73. Montambault JR, et al. 2015. Use of monitoring data to support conservation management and policy decisions in Micronesia. Conservation Biology 29:1279-1289.
74. Mountjoy NJ, Whiles MR, Spyreas G, Lovvorn JR, Seekamp E. 2016. Assessing the efficacy of community-based natural resource management planning with a multi-watershed approach. Biological Conservation 201:120-128.
75. Navarro MKD. 2015. Socio-Economic Scale (SES) as a measure of economic well-being: A case in Barangay Lumbia, Cagayan De Oro City, Philippines. Mediterranean Journal of Social Sciences 6:103-112.
76. Noss AJ, Oetting I, Cuéllar RL. 2005. Hunter self-monitoring by the Isoseño-Guaraní in the Bolivian Chaco. Biodiversity and Conservation 14:2679-2693.
77. Ohayon JL, Cousins E, Brown P, Morello-Frosch R, Brody JG. 2017. Researcher and institutional review board perspectives on the benefits and challenges of reporting back biomonitoring and environmental exposure results. Environmental Research 153:140-149.
78. Olendo MI, Okemwa GM, Munga CN, Mulupi LK, Mwasi LD, Mohamed HB, Sibanda M, Ong'Anda HO. 2019. The value of long-term, community-based monitoring of marine turtle nesting: A study in the Lamu archipelago, Kenya. ORYX 53:71-80.
79. Palmer Fry B. 2011. Community forest monitoring in REDD+: The 'M' in MRV? Environmental Science and Policy 14:181-187.

80. Paneque-Gálvez J, McCall MK, Napoletano BM, Wich SA, Koh LP. 2014. Small drones for community-based forest monitoring: An assessment of their feasibility and potential in tropical areas. Forests 5:1481-1507.
81. Peters CB, Zhan Y, Schwartz MW, Godoy L, Ballard HL. 2017. Trusting land to volunteers: How and why land trusts involve volunteers in ecological monitoring. Biological Conservation 208:48-54.
82. Peters MA, Eames C, Hamilton D. 2015. The use and value of citizen science data in New Zealand. Journal of the Royal Society of New Zealand 45:151-160.
83. Peters MA, Hamilton D, Eames C, Innes J, Mason NWH. 2016. The current state of community-based environmental monitoring in New Zealand. New Zealand Journal of Ecology 40:279-288.
84. Pilcher N, Chaloupka M. 2013. Using community-based monitoring to estimate demographic parameters for a remote nesting population of the Critically Endangered leatherback turtle. Endangered Species Research 20:49-57.
85. Pocock MJO, et al. 2018. A Vision for Global Biodiversity Monitoring With Citizen Science. Pages 169-223 in Bohan DA, Dumbrell AJ, Woodward G, Jackson M, eds. Advances in Ecological Research: Academic Press Inc.
86. Pratihast AK, DeVries B, Avitabile V, de Bruin S, Kooistra L, Tekle M, Herold M. 2014. Combining satellite data and community-based observations for forest monitoring. Forests 5:2464-2489.
87. Rae M, Miró A, Hall J, O'Brien K, O'Brien D. 2019. Evaluating the validity of a simple citizen science index for assessing the ecological status of urban drainage ponds. Ecological Indicators 98:1-8.
88. Ridder D, Pahl-Wostl C. 2005. Participatory Integrated Assessment in local level planning. Regional Environmental Change 5:188-196.
89. Scanlon BR, Ruddell BL, Reed PM, Hook RI, Zheng C, Tidwell VC, Siebert S. 2017. The food-energy-water nexus: Transforming science for society. Water Resources Research 53:3550-3556.
90. Scherber C, Beduschi T, Tscharntke T. 2018. Novel approaches to sampling pollinators in whole landscapes: a lesson for landscape-wide biodiversity monitoring. Landscape Ecology.
91. Seak S, Schmidt-Vogt D, Thapa GB. 2011. A comparison between biodiversity monitoring systems to improve natural resource management in Tonle Sap Biosphere Reserve, Cambodia. International Journal of Biodiversity Science, Ecosystem Services and Management 7:258-272.
92. ---. 2012. Biodiversity monitoring at the tonle sap lake of cambodia: A comparative assessment of local methods. Environmental Management 50:707-720.
93. Sharpe A, Conrad C. 2006. Community based ecological monitoring in Nova Scotia: Challenges and opportunities. Environmental Monitoring and Assessment 113:395-409.
94. Sheppard SA, Terveen L. 2011. Quality is a verb: The operationalization of data quality in a citizen science community. Pages 29-38.
95. Shi YX, Guéguen C. 2017. In Situ Monitoring of Labile Vanadium in the Mackenzie River Basin (Canada) Using Diffusive Gradients in Thin Films. Water, Air, and Soil Pollution 228 (art. 420).
96. Shirk JL, et al. 2012. Public participation in scientific research: A framework for deliberate design. Ecology and Society 17.

97. Siruma A, Tandlich R, Hornby D, Srinivas CS. 2015. Role of higher education institution and community engagement in disaster management in South Africa. Pages 783-789: International Multidisciplinary Scientific Geoconference.
98. Skutsch M, Turnhout E, Vijge MJ, Herold M, Wits T, Den Besten JW, Torres AB. 2014. Options for a national framework for benefit distribution and their relation to community-based and national REDD+ monitoring. Forests 5:1596-1617.
99. Staddon SC, Nightingale A, Shrestha SK. 2014. The Social Nature of Participatory Ecological Monitoring. Society and Natural Resources 27:899-914.
100. ---. 2015. Exploring participation in ecological monitoring in Nepal's community forests. Environmental Conservation 42:268-277.
101. Starkey E, Parkin G, Birkinshaw S, Large A, Quinn P, Gibson C. 2017. Demonstrating the value of community-based ('citizen science') observations for catchment modelling and characterisation. Journal of Hydrology 548:801-817.
102. Stone J, Barclay J, Simmons P, Cole PD, Loughlin SC, Ramón P, Mothes P. 2014. Risk reduction through community-based monitoring: the vigías of Tungurahua, Ecuador. Journal of Applied Volcanology 3 (art. 11).
103. Storey RG, Wright-Stow A. 2017. Community-based monitoring of New Zealand stream macroinvertebrates: agreement between volunteer and professional assessments and performance of volunteer indices. New Zealand Journal of Marine and Freshwater Research 51:60-77.
104. Storey RG, Wright-Stow A, Kin E, Davies-Colley RJ, Stott R. 2016. Volunteer stream monitoring: Do the data quality and monitoring experience support increased community involvement in freshwater decision making? Ecology and Society 21 (art. 32).
105. Strangway RE, Dunn M, Erless R. 2016. Monitoring Nûtimesânân following the diversion of our river: AcCommunity-led registry in Eeyou Istchee, Northern Québec. Journal of Environmental Assessment Policy and Management 18 (art. 1650001).
106. Stuart-Hill G, Diggle R, Munali B, Tagg J, Ward D. 2005. The event book system: A community-based natural resource monitoring system from Namibia. Biodiversity and Conservation 14:2611-2631.
107. Tapia A, Lalone N, MacDonald E, Case N, Hall M, Heavner M. 2014. Aurorasaurus: Citizen science, early warning systems and space weather. Pages 30-32: AI Access Foundation.
108. Topp-Jørgensen E, Poulsen MK, Lund JF, Massao JF. 2005. Community-based monitoring of natural resource use and forest quality in montane forests and miombo woodlands of tanzania. Biodiversity and Conservation 14:2653-2677.
109. Torres AB. 2014. Potential for integrating community-based monitoring into REDD+. Forests 5:1815-1833.
110. Townsend WR, Borman A R, Yiyoguaje E, Mendua L. 2005. Cofán Indians' monitoring of freshwater turtles in Zábalo, Ecuador. Biodiversity and Conservation 14:2743-2755.
111. Turreira-García N, Lund JF, Domínguez P, Carrillo-Anglés E, Brummer MC, Duenn P, Reyes-García V. 2018. What's in a name? Unpacking "participatory" environmental monitoring. Ecology and Society 23 (art. 24).
112. Turreira-García N, Meilby H, Brofeldt S, Argyriou D, Theilade I. 2018. Who wants to save the forest? Characterizing community-led monitoring in Prey Lang, Cambodia. Environmental Management 61:1019-1030.

113. Uychiaoco AJ, Arceo HO, Green SJ, De La Cruz MT, Gaite PA, Aliño PM. 2005. Monitoring and evaluation of reef protected areas by local fishers in the Philippines: Tightening the adaptive management cycle. Biodiversity and Conservation 14:2775-2794.
114. Vani BC, Shimabukuro MH, Galera Monico JF. 2017. Visual exploration and analysis of ionospheric scintillation monitoring data: The ISMR Query Tool. Computers and Geosciences 104:125-134.
115. Verbrugge LNH, Ganzevoort W, Fliervoet JM, Panten K, van den Born RJG. 2017. Implementing participatory monitoring in river management: The role of stakeholders' perspectives and incentives. Journal of Environmental Management 195:62-69.
116. Verstraete MM, Hutchinson CF, Grainger A, Stafford Smith M, Scholes RJ, Reynolds JF, Barbosa P, Léon A, Mbow C. 2011. Towards a global drylands observing system: Observational requirements and institutional solutions. Land Degradation and Development 22:198-213.
117. Villaseñor E, Porter-Bolland L, Escobar F, Guariguata MR, Moreno-Casasola P. 2016. Characteristics of participatory monitoring projects and their relationship to decision-making in biological resource management: a review. Biodiversity and Conservation 25:2001-2019.
118. Whitelaw G, Vaughan H, Craig B, Atkinson D. 2003. Establishing the Canadian community monitoring network. Environmental Monitoring and Assessment 88:409-418.
119. Williams P, Alessa L, Abatzoglou JT, Kliskey A, Witmer F, Lee O, Trammell J, Beaujean G, Venema R. 2018. Community-based observing networks and systems in the Arctic: Human perceptions of environmental change and instrument-derived data. Regional Environmental Change 18:547-559.
120. Wilson NJ, Mutter E, Inkster J, Satterfield T. 2018. Community-based monitoring as the practice of Indigenous governance: A case study of Indigenous-led water quality monitoring in the Yukon River Basin. Journal of Environmental Management 210:290-298.
121. Wiseman ND, Bardsley DK. 2016. Monitoring to learn, learning to lonitor: A critical analysis of opportunities for Indigenous community-based monitoring of environmental change in Australian Rangelands. Geographical Research 54:52-71.
122. Wofford P, Segawa R, Schreider J, Federighi V, Neal R, Brattesani M. 2014. Community air monitoring for pesticides. Part 3: Using health-based screening levels to evaluate results collected for a year. Environmental Monitoring and Assessment 186:1355-1370.
123. Yang ES, Christopher SA, Kondragunta S, Zhang X. 2011. Use of hourly Geostationary Operational Environmental Satellite (GOES) fire emissions in a Community Multiscale Air Quality (CMAQ) model for improving surface particulate matter predictions. Journal of Geophysical Research Atmospheres 116 (art. D04303).
124. Zapponi L, et al. 2017. Citizen science data as an efficient tool for mapping protected saproxylic beetles. Biological Conservation 208:139-145.
