# Supplementary material for: Connecting Top-Down and Bottom-Up Approaches in Environmental Observing
Source: Bioscience. 2021 Apr 28;71(5):467–83. doi: 10.1093/biosci/biab018 (PMC8106998; doi:10.1093/biosci/biab018)
Supplement: biab018_Supplemental_Files [file biab018_supplemental_files.zip › Eicken_BioScience_Supplement_CBMPractitionerSurvey.pdf]

CBM Practitioner Questionnaire

The CBM practitioners in our survey were asked general questions relevant for all Arctic monitoring systems, including both scientist-executed and community-based monitoring programs, and questions of particular relevance to CBM programs. The general questions were about the respondent, the general characteristics of the attributes observed, the sustainability of the monitoring, and the use and management of the data (see "Questionnaire A", available at https://intaros.nersc.no/node/651). The questions of particular relevance to CBM programs are presented below.

Questionnaire: Community-Based Monitoring Program

Section I: Central questions

Section II: General information

Section III: Community members

Section IV: The data

I. Central Questions

1. What is the aim of the monitoring program?

(Examples: To protect rights over land and resources; To ensure sustainable use of resources; To protect threatened biota; To obtain a better understanding of the environment; Monitoring is just part of "everyday life"; Other)

2. Who do you consider to be the users of the data/results from the monitoring program?

Write who you believe/ know make use of the data.

Other community members

Government agency

Civil society organisations

Academic institutions

Do not know

Other: _________________

3. Does the monitoring program link to natural resource governance (management of the resources), or to scientific research?

Link to natural resource governance

Link to scientific research

No links to natural resource governance or scientific research

Do not know

Other__________________

4. Do you supply or pass on your monitoring data to other organisations?

Yes

No

No, but we would like to

Do not know

Other___________

5. If yes, to whom?

Select all you agree with.

Government agency

Civil society organisation / NGO

Academic institution

Other:____________

6. Has the monitoring contributed to the local community—and how?

Positively. Development of pride or self-esteem (COGNITIVE EMPOWERMENT)

Positively. Participation in decision-making, increased local governance (POLITICAL EMPOWERMENT)

Positively. Education, or improvement of local organizations (SOCIAL EMPOWERMENT)

Positively. Financial resources, increased control of subsistence resources (ECONOMIC EMPOWERMENT)

Positively, in OTHER WAYS (explain under Question 9)

Negatively (explain under Question 9)

Do not know

Other________________

7. What are the sources of financial support?

Government agency

Civil society organisation / NGO / community-based organization

Private foundation

Academic institution

Do not know

Other____________

8. Which stages of the monitoring process were the community members and external agents (scientists, government staff) involved in?

Community members: the DESIGN of the monitoring system

Community members: the DATA COLLECTION in the monitoring system

Community members: The DATA INTERPRETATION in the monitoring system

Community members: The USE OF THE RESULTS from the monitoring system

External agents: the DESIGN of the monitoring system

External agents: the DATA COLLECTION in the monitoring system

External agents: The DATA INTERPRETATION in the monitoring system

Do not know

Other_____________

9. Anything else you want to add to the questions in this section?

II. General Information

10. Which attributes are monitored by the monitoring program?

Select all you agree with.

WILDLIFE (animals)

Insects

Shellfish

Fish

Birds

Mammals

Other species

VEGETATION

Fungi

Plants

ABIOTIC PHENOMENA

Water

Air

Snow

Ice

Wind

Weather

Contaminants

Other abiotic phenomena

SOCIO-CULTURAL ATTRIBUTES

Human health

Wellness

Language

Traditional knowledge transmission

Other socio-cultural attributes

Other____________

11. What landscape type is monitored by the monitoring program?

Taiga or boreal forest

Tundra

Freshwater

Coastal

Sea

Other_____________

12. Who decided on what and where data should be collected?

Scientists

Government staff

Community members

Other____________

13. Briefly describe the methodology (one sentence only, please)

14. Do you use some kind of measure of effort?

For example, number of hunting trips, hooks and net used?

Yes

No

Do not know

15. Equipment needs for data collection: How much does the equipment required by one data collector cost?

0 -100 $

100 -1000 $

> 1000 $

Do not know

16. What is the frequency of data collection?

Indicate the intervals between successive bouts of data collection

Daily

Weekly

Monthly

Quarterly (three-monthly)

Yearly

Do not know

Other__________

17. Is monitoring done during certain time of year?

No distinct period

January

February

March

April

May

June

July

August

September

October

November

December

Do not know

18. Anything else you want to add to the questions in this section?

III. Community Members

19. How many community members participate in total in the monitoring process?

Select one of the available choices.

0–10

11–100

101–500

501–1000

>1000

Do not know

20. What is the proportion of women?

Select one of the available choices.

0 %

1–5%

6–25%

26–75%

76–100%

Do not know

21. What age classes are included in the monitoring process?

Children (0–18)

Youth (19–26)

Adults (27–60)

Elders (>60)

Do not know

22. How were the community members chosen?

Appointed by somebody based on their background

They did themselves propose their involvement

Do not know

Other_______________

23. What are the sources of motivation for community members to participate in the monitoring system?

Have their voices heard / protect rights over land, sea and resources

Leisure interest / socializing

Social engagement

Personal benefits

Mandatory

Suatain health and abundance of wildlife

Do not know

Other_____________

24. Do the community members get compensation/salary for being involved in the monitoring program?

Yes

No

Do not know

25. Do community members obtain feed-back on the findings from the monitoring?

Select one of the available choices.

Yes

No

Do not know

26. Anything else you want to add to the questions in this section?

IV. The Data

27. Are there data validation processes built into the monitoring program?

For example by triangulation (cross checking)

No inbuilt validation processes

Yes, triangulation across COMMUNITY MEMBERS

Yes, triangulation across COMMUNITIES

Yes, triangulation across METHODS

Yes, other types of validation processes (not triangulation)

Do not know

Other___________

28. Is the data quality of the collected data being checked?

For example data spreadsheets can be checked for data encoding errors, anomalies and data that are beyond expected range, before the data is used. Select one of the available choices.

Yes

No

Do not know

Other________

29. What language is the original data in?

Select one of the available choices.

Local dialect (not the national language)

The national language

English

Do not know

Other_____________

30. How long after data collection is the data available to users

Select one of the available choices.

Data are accessible after an unknown period,

Data are accessible some years after acquisition

Data are accessible within 6–12 months after acquisition

Data are accessible within a month after acquisition

Data are accessible within a week after acquisition

Data are accessible within a day after acquisition

Data are accessible within 3 hours after acquisition

Data are accessible in real time

Do not know

Other________________

31. Has any assessment of the program been undertaken within the last 3 years?

Select one of the available choices.

Yes

No

Do not know

32. Principal challenges to the monitoring program?

Select all you agree with.

Limited funding

Personal hardship

Violation of intellectual property rights/Free Prior and Informed Consent

Political challenges

Fatigue among community members

Do not know

Other_____________

33. Anything else you like to add about the monitoring program?

34. Your name (the encoder of the metadata)

35. Your email address
