# Supplementary material for: Connecting Top-Down and Bottom-Up Approaches in Environmental Observing
Source: Bioscience. 2021 Apr 28;71(5):467–83. doi: 10.1093/biosci/biab018 (PMC8106998; doi:10.1093/biosci/biab018)
Supplement: biab018_Supplemental_Files [file biab018_supplemental_files.zip › Eicken_BioScience_Supplement_RespondentstoSurvey.pdf]

Arctic CBM programs that provided information for our survey

Title	Link	Content
A-OK (Alaska Arctic Observatory and Knowledge Hub)	https://eloka-arctic.org/sizonet/	Access to all data via database search function
Arctic and Earth SIGNs	https://www.globe.gov/web/arctic-and-earth-signs	Program information
Arctic Borderlands	https://www.arcticborderlands.org	Program information about Arctic Borderlands Ecological Knowledge Society and link to guidelines for requesting access to data
Bird phenology	https://dataverse.no/dataverse/uit	Large amount of open research data including bird phenology data.
	https://dataverse.no/dataset.xhtml?persistentId=doi:10.18710/4MCRQL	Data on first arrival dates of spring migrants
BuSK (Building Shared Knowledge)	https://katersaatit.wordpress.com/dyr-og-fangst/	Program information in Greenlandic and Danish language
	https://asiaq.maps.arcgis.com/apps/View/index.html?appid=72a0b7241ef341b0af3fb3812eddb320	Access to data

CSIPN (Centre for Support to Indigenous Peoples of the North)	https://www.uarctic.org/member-profiles/russia/8440/centre-for-support-of-indigenous-peoples-of-the-north-russian-indigenous-training-centre	Program information in English language. Use http://www.csipn.ru for additional information in Russian language.
Evenk & Izhma Peoples	N/A	
Fávllis	http://site.uit.no/favllis/	Program information, as well as Indigenous and local knowledge, in Norwegian.
	https://www.meron.no/nb/	Data from Fávllis are/will become available via this link.
FMI (Finnish Meteorological Institute) Snow Depth	http://globefinland.fi/glofin/projektit.html	Program information in Finish, with link to data in English, as well as information on FMI snow depth measurement campaign.
Fuglavernd	https://fuglavernd.is/verkefnin/gardfuglar/gardfuglahelgi/	Program information in Icelandic.

George River	N/A	
Farmers and Herders	See "Summer Farmers and Small Herders"	
Hares	haran.fo	Harvest data including graphics, in Faroese
ION (Indigenous Observation Network) Yukon River	https://www.yritwc.org/science	Program information in English
	https://www.sciencebase.gov/catalog/item/573f3b8de4b04a3a6a24ae28	Water quality data example:
Local Environmental Observer	https://www.leonetwork.org/en/docs/about/about	Program information in English
	https://www.leonetwork.org/en/	Access to data
Marion Watershed	N/A	
Nordland Ærfugl	http://www.eiderducks.no/?side=om-nordland-aerfugllag&language=no	Program information in Norwegian
Oral History	http://www.snowchange.org/efforts-in-the-skolt-sami-areas-of-	Program information in English

	naatamo-watershed-finland/collaborative-management-along-the-naatamo-watershed/	
	http://www.snowchange.org/pages/wp-content/uploads/2015/09/Snowchange-Discussion-Paper-9.pdf	Example of available reports
Pilot Whale	https://heimabeiti.fo/hagtol	Harvest data from 1584 to 2020 in Faroese
Piniarneq	https://www.sullissivik.gl/Emner/Jagt_Fangst_og_Fiskeri/Jagtbevis/Fritidsjagtbevis_samlet?	Links to Piniarneq harvest reporting only (Greenlandic and Danish). No information about how to get data, but data is likely to be available upon request.
PISUNA	http://www.pisuna.org/uk_index.html	Program information in Greenlandic, Danish, and English
	https://eloka-arctic.org/pisuna-net/en/	Searchable database
Renbruksplan	http://www.renbruksplan.se	Program information in Swedish and local language
	https://www.sametinget.se/116236	Information on database under construction in Swedish and local language
River Owners Iceland	http://www.angling.is/en/catch-statistics/	Link to data in English and Icelandic

Sea Ice for Walrus Outlook	https://www.arcus.org/siwo	Program information in English
Seal Monitoring	https://selasetur.is/en/research/557-2/	Program information in English and Icelandic
Summer Farmers and Small Herders	https://www.slu.se/en/Collaborative-Centres-and-Projects/swedish-biodiversity-centre1/Research/projects/ongoing-phd-projects-/	Some program information in English
Walrus Haulout Monitoring	N/A	
Wildlife Triangles	https://www.nrcresearchpress.com/doi/pdf/10.1139/cjfr-2015-0454	Program information in English
Wild North	http://rannsoknasetur.hi.is/university_icelands_research_center_husavik	General information on the research center
WinterBerry	https://sites.google.com/alaska.edu/winterberry/	Program information in English
Älgdata	http://www.algdata.se/Sv/Pages/default.aspx	Program information in Swedish

http://www.algdata.se/Sv/statistik/Pages/default.aspx	Link to data in Swedish
