# Supplementary material for: Connecting Top-Down and Bottom-Up Approaches in Environmental Observing
Source: Bioscience. 2021 Apr 28;71(5):467–83. doi: 10.1093/biosci/biab018 (PMC8106998; doi:10.1093/biosci/biab018)
Supplement: biab018_Supplemental_Files [file biab018_supplemental_files.zip › Eicken_BioScience_Supplement_ReviewSearchCriteriaMethods.pdf]

Selection method for literature-reviewed studies

We conducted a search on Scopus (an abstract and citation database that is operated by Elsevier; https://service.elsevier.com/app/home/supporthub/scopus/) using the following keywords and delimiters:

KEY (community AND based AND (monitoring OR observ*)) AND (EXCLUDE (SUBJAREA , "MEDI")) AND (EXCLUDE (SUBJAREA , "MATH") OR EXCLUDE (SUBJAREA , "NURS") OR EXCLUDE (SUBJAREA , "PHYS") OR EXCLUDE (SUBJAREA , "PHAR") OR EXCLUDE (SUBJAREA , "PSYC") OR EXCLUDE (SUBJAREA , "BUSI") OR EXCLUDE (SUBJAREA , "MATE") OR EXCLUDE (SUBJAREA , "HEAL") OR EXCLUDE (SUBJAREA , "DENT"))

The initial search yielded 549 results, which were then manually analyzed for subject matter relevance. The final 124 papers were selected by first determining relevance by title. The papers that had clear relevance had the following keywords/terms:

1. Community-based monitoring
2. Community-based environmental monitoring
3. Community-based observation(s)
4. Community monitoring
5. Citizen science
6. Monitoring systems
7. Participatory … monitoring
8. Monitoring…community based…management
9. Monitoring…locally-based approaches

In instances where titles hinted rather than showed clear relevance to the subject matter, we read abstracts for further clarification. Most papers whose abstracts were examined but ultimately deemed irrelevant dealt with a) Community Based Participatory Research (CBPR) which focused on inclusive research to address social science or medical issues, b) multi-platform methods employed in computer science research, and c) community-based co-management of natural resources.

Those that turned out to be relevant had the following terms/keywords in their titles that hinted at non-expert involvement in community-based monitoring/observation:

1. "Cost effective practices…monitoring"
2. "Social Network … community environmental awareness"
3. "Mobile stream sampling"
4. "… sustainable use and management of Arctic species"
5. Assessing and monitoring…using object-based image and multiple endmember….
6. "…open source … processing weather radar data
7. "Global … observing system"
8. "Using community members to assess …. Fisheries"

In the table below, we identify the region and country of origin for each study, show which utilized top-down versus bottom down methods, and which entailed elements of local and Indigenous knowledge and citizen science. We also provide the keywords used in each study, where available, as well as abstracts and weblinks to their location. We also indicate whether the projects fall into the local, regional, or global categories.

From Scopus references (n=124):

ILK only	Citizen science only	Both ILK and Citizen science	Citizen science Total
18	32	2	34
