# Supplementary material for: Connecting Top-Down and Bottom-Up Approaches in Environmental Observing
Source: Bioscience. 2021 Apr 28;71(5):467–83. doi: 10.1093/biosci/biab018 (PMC8106998; doi:10.1093/biosci/biab018)
Supplement: biab018_Supplemental_Files [file biab018_supplemental_files.zip › Manuscript ID-BIOS-19-0331-R2 Figure reproduction.pdf]

Bioscience submission
Manuscript ID: BIOS-19-0331.R2
Manuscript Title: Connecting Top-Down and Bottom-Up Approaches in Environmental Observing
Authors, Eicken, Danielsen et al.

The manuscript BIOS-19-0331.R2 reproduces a figure (our Fig. 3) from

Evangelista, P. H., Mohamed, A. M., Hussein, I. A., Saied, A. H., Mohammed, A. H., and Young, N. E.. 2018. Integrating indigenous local knowledge and species distribution modeling to detect wildlife in Somaliland. *Ecosphere* 9(3):e02134. 10.1002/ecs2.2134

https://esajournals.onlinelibrary.wiley.com/doi/10.1002/ecs2.2134

Ecosphere is an open access journal that allows for reproduction of articles as in our case: https://esajournals.onlinelibrary.wiley.com/hub/journal/21508925/resources/author-guidelines-ECS2#Copyright_and_Open_Access_Agreement

Below follows the explicit permission with additional information from the publisher’s website.

Copyright Clearance Center RightsLink®

Help Email Support

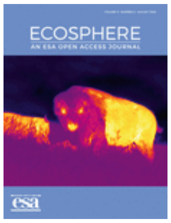

Integrating indigenous local knowledge and species distribution modeling to detect wildlife in Somaliland

Author: Nicholas E. Young, Abdikadir H. Mohammed, Abdinasir H. Saied, et al

Publication: Ecosphere

Publisher: John Wiley and Sons

Date: Mar 12, 2018

© 2018 Evangelista et al.

Open Access Article

This is an open access article distributed under the terms of the Creative Commons CC BY license, which permits unrestricted use, distribution, and reproduction in any medium, provided the original work is properly cited.

You are not required to obtain permission to reuse this article.

For an understanding of what is meant by the terms of the Creative Commons License, please refer to Wiley's Open Access Terms and Conditions.

Permission is not required for this type of reuse.

Wiley offers a professional reprint service for high quality reproduction of articles from over 1400 scientific and medical journals. Wiley's reprint service offers:

- Peer reviewed research or reviews
- Tailored collections of articles
- A professional high quality finish
- Glossy journal style color covers
- Company or brand customisation
- Language translations
- Prompt turnaround times and delivery directly to your office, warehouse or congress.

Please contact our Reprints department for a quotation. Email corporatesaleseurope@wiley.com or corporatesalesusa@wiley.com or corporatesalesDE@wiley.com.

© 2021 Copyright - All Rights Reserved | Copyright Clearance Center, Inc. | Privacy statement | Terms and Conditions
Comments? We would like to hear from you. E-mail us at customercare@copyright.com
